# Seasonal change is a major driver of soil resistomes at a watershed scale

**DOI:** 10.1038/s43705-021-00018-y

**Published:** 2021-05-20

**Authors:** Qian Xiang, Min Qiao, Dong Zhu, Madeline Giles, Roy Neilson, Xiao-Ru Yang, Yong-Guan Zhu, Qing-Lin Chen

**Affiliations:** 1 State Key Laboratory of Urban and Regional Ecology, Research Center for Eco-Environmental Sciences, Chinese Academy of Sciences, Beijing, China; 2 University of Chinese Academy of Sciences, Beijing, China; 3 Ecological Sciences, The James Hutton Institute, Dundee, Scotland UK; 4 Key Lab of Urban Environment and Health, Institute of Urban Environment, Chinese Academy of Sciences, Xiamen, China; 5 Faculty of Veterinary and Agricultural Sciences, The University of Melbourne, Parkville, VIC Australia

**Keywords:** Microbial ecology, Microbial ecology, Biogeography

## Abstract

Soils harbor the most diverse naturally evolved antibiotic resistomes on Earth that threaten human health, ecosystem processes, and food security. Yet the importance of spatial and temporal variability in shaping the distribution of soil resistomes is not well explored. Here, a total of 319 topsoil samples were collected at a watershed scale during four seasons (spring to winter) and high-throughput quantitative PCR (HT-qPCR) was used to characterize the profiles of soil antibiotic resistance genes (ARGs). A significant and negative correlation was observed between soil ARG profiles and seasonal dissimilarity, which along with seasonally dependent distance-decay relationships highlight the importance of seasonal variability in shaping soil antibiotic resistomes. Significant, though weak, distance-decay relationships were identified in spring, summer and winter, for ARG similarities with geographic distances. There were also strong interactions between specific soil ARGs and Actinobacteria, Firmicutes and Proteobacteria. Moreover, we found that the relative abundance of soil Actinobacteria, Firmicutes and Proteobacteria correlated significantly with annual mean temperature and annual mean precipitation at a watershed scale. A random forest model showed that seasonal change rather than spatial variation was the most important predictor of the composition of soil ARGs. Together, these results constitute an advance in our understanding of the relative importance of spatial and temporal variability in shaping soil ARG profiles, which will provide novel insights allowing us to forecast their distribution under a changing environment.

## Introduction

Antibiotic resistance is a natural phenomenon used by bacteria to withstand the negative effects of antibiotics released by other organisms.^1,2^ However, the dissemination of antibiotic resistance genes (ARGs) throughout the environment has been accelerated by extensive anthropogenic activities.^3–7^ Consequently, increased occurrence and an accelerating spread of ARGs in bacterial pathogens has been one of the major threats to human, animal and plant health and food safety.^8^

Soils are an important reservoir for ARGs and constitute an important habitat for the exchange of ARGs among bacteria, including some major clinical pathogens.^9^ The continuous and intensive application of organic fertilizers and use of wastewater irrigation has led to a significant increase in the background levels of antimicrobial resistance in soils, therefore enhancing the likelihood of wider dissemination of antibiotic resistant bacteria (ARB) via surface runoff, wind, soil animal and plant.^3,10–13^ For example, in peri-urban areas, where food production occurs, ARBs in soil could directly enter the food chain via consumption of contaminated crops grown in manure-fertilized soil and subsequently impact human health.^14–16^ Recently, several studies have investigated the distribution, fate and ecological drivers of ARGs from local to global scales.^3,10,17–19^ For instance, a continental scale investigation that profiled ARGs in sediments from 18 estuaries, representing 4000 km of coastal China, found that human activity was responsible for the abundance and dissemination of ARGs.^17^ At global scale, Bahram et al.^20^ found that the relative abundance of ARGs in topsoil was strongly related to fungal biomass and the bacterial/fungal biomass ratio. By comparison, the interaction between temporal changes and spatial variability of ARGs has received less attention. Most previous studies have overlooked potential temporal and spatial effects often using samples collected from a single point in time.^18,21^ Studies that have explored temporal variation in soil ARGs provided critical knowledge on temporal and ecological drivers of ARG patterns.^22–24^ However, many of these studies were based at microcosm scale under controlled conditions, which may not accurately predict antibiotic resistance under human disturbance and environmental change.

Here, the objective of this study was to investigate spatio-temporal dynamics of soil ARGs, and to evaluate the relative importance of space and time on shaping soil ARG profiles. To achieve this, 319 topsoil samples were collected in peri-urban areas across the Zhangxi watershed during four seasons (spring, summer, autumn and winter) with high-throughput quantitative PCR (HT-qPCR) used to quantify the abundance of 296 ARGs in order to create soil ARG profiles. While agronomic management (e.g., fertilization, crops) could be an important driver of soil ARGs, seasonal variability will be a comprehensive index of the (a)biotic changes, therefore we hypothesize that seasonal variability will be the major driver that determines ARG abundance and composition and will be more important than geographical location at a watershed scale.

## Materials and methods

### Sampling site and sample collection

The study area comprised of 83 sampling sites covering a single watershed (Zhangxi, 28.85–30.55 N, 120.92–122.27 E), covering an area of 85 km^2^ and an elevation range of 3–763 m, in the peri-urban area of Ningbo, Zhejiang Province, southeast China. The watershed is a mixed land-use watershed, including forests (natural secondary forest, managed forest), farmland (vegetable fields, nursery) and orchards (Supplementary Fig. S1). Zhangxi watershed has a subtropical monsoon climate, warm, humid and windy during April–September, and the rainy season typically commences in June. Topsoil (0–20 cm) samples (*n* = 319) were collected in April, July, November 2017 and January 2018, representing the spring, summer, autumn and winter seasons. After removing plant debris and stones, at each sampling site seven soil cores were randomly taken, pooled, and thoroughly mixed. After collection, soil samples were immediately frozen with dry ice, transported to the laboratory and stored at −20 °C until DNA extraction.

### Soil DNA extraction, amplification, Illumina sequencing

Soil DNA was extracted from 0.5 g of soil using a FastDNA^®^ Spin Kit for soil (MP Biomedical, Santa Ana, California, USA). DNA concentration and quality were checked using a Qubit 3.0 (Thermo Fisher Scientific, USA) and gel electrophoresis with a 1.0% agarose gel. Thereafter, DNA extracts were stored at −20 °C until further analyses.

The primer pair F515 (5′-GTGCCAGCMGCCGCGG-3′) and R907 (5′-CCGTCAATTCMTTTRAGTTT-3′) was used to amplify the 16S V4-V5 region.^25^ Each sample was amplified in a 50 µl PCR reaction mixture with 25 µl TaKaRa ExTaq master mix, 0.5 µl bovine serum albumin (BSA), 1 µl of each forward and reverse primer, 1 µl DNA template and 21.5 µl nuclease-free PCR-grade water. PCR reaction conditions consisted of a 5 min initial enzyme activation at 95 °C, followed by 30 cycles of denaturation at 95 °C for 30 s, annealing at 58 °C for 30 s and extension at 72 °C for 30 s, with a final extension at 72 °C for 5 min. Negative controls (template DNA replaced with water) were included for detection of any potential contamination during PCR. All PCR products were purified using a Wizard SV Gel and PCR Clean-Up System (TIANGEN Biotech, Beijing, China). Purified PCR products were quantified and pooled together for Illumina Hiseq2500 sequencing (Novogene, Beijing, China). Raw pair-end reads were filtered to discard raw reads containing three or more ambiguous nucleotides, reads with low (<20) average quality scores and those with short reads (<100 nt). The QIIME1.9 pipeline was used to process and analyze pre-processed raw sequences.^26^ Operational taxonomic units (OTUs) were identified using the UCLUST algorithm with a phylotype defined at 97% sequence similarity.^27^ Chimeric sequences, chloroplast and mitochondrial OTUs (around 1%) and singleton OTUs were discarded from the final OTU data set. Taxonomic classification was conducted using the Ribosome Database Project Classifier with a confidence threshold of 0.80,^28^ against the SILVA database.^29^ Raw sequences were deposited in the National Center for Biotechnology Information Sequence Read Archive under the accession number (PRJNA718353).

### High-throughput quantitative PCR (HT- qPCR)

Soil ARG profiles were characterized using DNA extracts and high-throughput qPCR with the Wafergen SmartChip Real-time PCR system (Warfergen Inc. USA).^6,30^ This system has previously been used for ARG detection in a range of samples including manure, sediment and plants. To ensure reproduceable and reliable data, compared to the original protocol,^6^ HT-qPCR conditions were optimized for soils. A total of 296 primer sets (Supplementary Table S1) were used, including 283 primer sets targeting almost all major classes of ARGs, 8 transposase genes and 4 integron-integrase genes. Before running HT-qPCR, DNA extracts were diluted to the same concentration, and BSA added to the PCR mixture to reduce potential inhibition. The following program was used for HT-qPCR amplification: initial enzyme activation at 95 °C for 10 min, thereafter 40 cycles of the following used for amplification: denaturation at 95 °C for 30 s and annealing at 60 °C for 30 s. Melt curves were automatically generated by Wafergen software. In addition, qPCR data were analyzed using SmartChip qPCR Software. Wells with multiple melting peaks or with amplification efficiencies beyond the range 1.8–2.2 were discarded. The relative copy number of ARGs and mobile genetic elements (MGEs) were calculated according to Ouyang et al.^31^

### Statistical analysis

All statistical analyses were conducted in the R3.6.1 environment (http://www.r-project.org/). Differences were considered significant at *P* < 0.05. In this study, overall patterns of ARGs and bacterial communities were determined by calculating dissimilarity matrixes using Bray–Curtis distances and compared between seasons and locations using non-metric multidimensional scaling analysis (nMDS) and PERMANOVA^30^ with the “labdsv”^32^ and “vegan” packages.^33^ Mantel tests were used to explore associations between bacterial communities and ARGs profiles and were conducted using the “vegan” package. In the present study, the values 1, 2, 3 and 4 were used to represent the spring, summer, autumn and winter, respectively, for further data analysis. The seasonal decay relationships were calculated using an ordinary least squares (OLS) model between seasonal dissimilarity (Euclidean distance) and ARG and bacterial community similarity (based on 1 – [dissimilarity of the Bray–Curtis distance metric]).^34^ We used a neutral community model (NCM)^35^ and normalized stochasticity ratio (*NST*)^36^ to determine the potential importance of stochastic and deterministic processes on ARG compositions and bacterial community assembly. The main predictors for the ARG profiles at the watershed scale were identified by a classification random forest (RF) analysis. The latitude and longitude data of each site (Supplementary Table S2) were transferred to rectangular data to represent spatial distance by function principal coordinates of neighboring matrices (pcnm) in R with the “vegan” package. In the RF model, pcnm1 served as spatial predictors for the ARG profile index, while the values 1, 2, 3 and 4 were served as temporal predictors for the ARG profile index. Bacteria at the phylum level were used to represent the biological predictors for ARG profiles. Network analysis was generated in the R3.6.1 environment using the “psych” package,^37^ to investigate the co-occurrence patterns between ARG subtypes and microbial taxa. Network graphs were visualized in Cytoscape 3.7.1 with a circular layout algorithm^38^ based on strong (Spearman’s *R*^2^ > 0.6) and significant (*P* < 0.001) correlations. Finally, we hypothesized that non-random co-occurrence patterns between ARGs and microbial taxa could indicate possible host information of ARGs if the ARGs and the co-existing microbial taxa possessed a strong and significantly positive correlation. OLS regression was conducted to test the relationships between the microbial taxa and annual mean temperature and annual mean precipitation (Supplementary Table S2). All boxplots, bar charts, scatter diagrams and OLS regression were generated using R with the “ggplot2” package,^39^ meanwhile, pairwise Wilcox tests of soil ARGs and bacterial Shannon index in spring, summer, autumn and winter were conducted with the “ggpubr”^40^ and “ggsignif”^41^ package.

## Results

### Diversity and abundance of ARGs in soil at a watershed scale

**Fig. 1:**
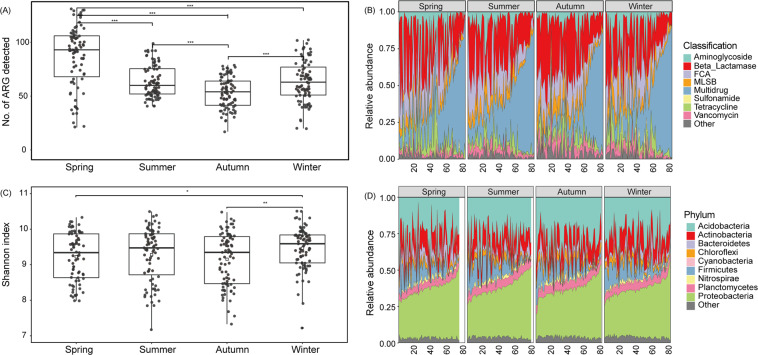
Diversity and abundance of soil ARGs and bacterial community members. (**A**) Boxplots represent the number of ARGs detected in spring, summer, autumn and winter; (**B**) Relative abundance of soil ARGs at watershed scale; (**C**) Boxplots represent bacterial Shannon index in spring, summer, autumn and winter; (**D**) Relative abundance of different phyla found in soil bacterial communities. The continuous numbers on plots (**B**) and (**D**) represent the 83 sampling sites. Significance levels are as follows: *P < 0.05, **P < 0.01, ***P < 0.001.

A total of 193 ARGs and 9 MGEs including 8 transposase genes and 1 *intI*-1(clinic) integron-integrase gene were detected from the watershed soil samples. Numbers of detected ARGs ranged from 21 to 131 in spring, 41 to 99 in summer, 17 to 79 in autumn, 20 to 102 in winter (Fig. 1A). These ARGs, represent almost all major classes of antibiotics commonly administered to humans and animals.

The number of ARGs detected in spring was significantly higher than all other seasons (Fig. 1A). The relative abundance of ARGs ranged from 6.50E-05 to 1.067 copies per 16S rRNA gene in spring, 0.012–0.136 copies per 16S rRNA gene in summer, 0.009–0.242 copies per 16S rRNA gene in autumn, and 0.005–0.928 copies per 16S rRNA gene in winter (Supplementary Fig. S2). By comparison, autumn had significantly lower abundances of ARGs than the other three seasons (*P* < 0.05). In addition, different types of ARGs showed distinct temporal patterns. For example, spring samples had a significantly greater abundance of Aminoglycoside resistance genes than the other seasons (*P* < 0.05), whereas autumn had the most Vancomycin resistance genes (*P* < 0.05). Genes resistant to fluoroquinolone, quinolone, florfenicol, chloramphenicol, and amphenicol (FCA) showed no significant temporal variation (*P* < 0.05) (Fig. 1B).

### Bacterial community composition in soil at a watershed scale

Across all samples, we obtained a total of 23,922,016 high quality bacterial sequences, which were grouped into 21,269 OTUs. Bacterial alpha-diversity (Shannon index) showed that winter soil samples had a more diverse bacterial community than was found in spring and autumn (*P* < 0.05) but not in summer (Fig. 1C). The soil microbial community was represented by 52 phyla, and was dominated by Actinobacteria, Acidobacteria, Bacteroidetes, Chloroflexi, Cyanobacteria, Firmicutes, Nitrospirae, Planctomycetes and Proteobacteria (Fig. 1D). Proteobacteria and Acidobacteria were the most dominant phyla, accounting for 35.1–82.5% of the total bacterial 16S rRNA gene sequences in spring, 34.3–86.1% in summer, 36.7–82.0% in autumn, and 37.0–78.1% in winter. By comparison, the relative abundance of Proteobacteria and Acidobacteria was significantly lower in winter than in spring, summer and autumn, whereas Actinobacteria, Firmicutes and Nitrospirae did not vary between seasons (*P* < 0.05) (Fig. 1D).

### Seasonal and spatial variation in soil microbiome at a watershed scale

Non-metric multidimensional scaling (nMDS) ordinations indicated that seasonal change shifted the overall profile of soil ARGs (Stress = 0.1884, *P* < 0.01) (Supplementary Fig. S3A). Similarly, soil bacterial community composition differed significantly between seasons, in particular, the bacterial community in spring separated from other seasons (Stress = 0.1469, *P* < 0.01) (Fig. S3B). A significant correlation was identified between the relative abundance of ARGs and seasonal dissimilarity (*R*^2^ = 0.0269, *P* < 0.001) (Supplementary Fig. S4A). Moreover, a negative relationship was observed between bacterial community similarity and seasonal dissimilarity (OLS analysis, *R*^2^ = 0.0701, *P* < 0.001) (Supplementary Fig. S4B).

**Fig. 2:**
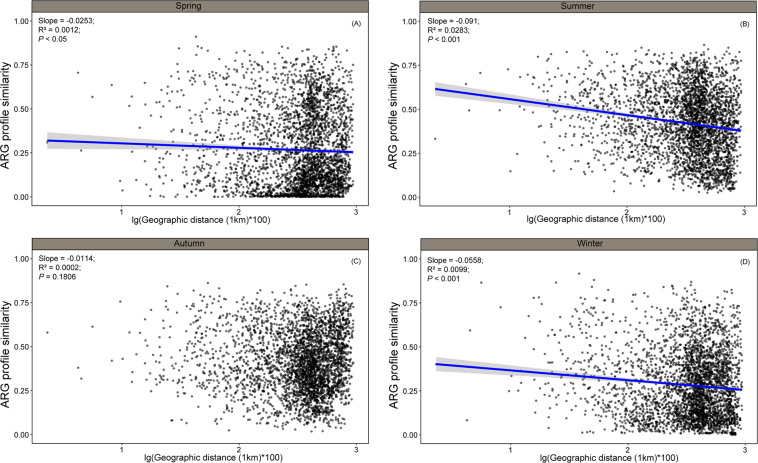
Spatial decay patterns of soil resistomes. Distance-decay relationships between ARG profile similarities and the geographic distances between sampling sites in (**A**) spring, (**B**) summer, (**C**) autumn and (**D**) winter. Similarity was calculated based on 1 – [dissimilarity of the Bray–Curtis distance metric]. Solid lines denote the ordinary least-squares linear regressions.

**Fig. 3:**
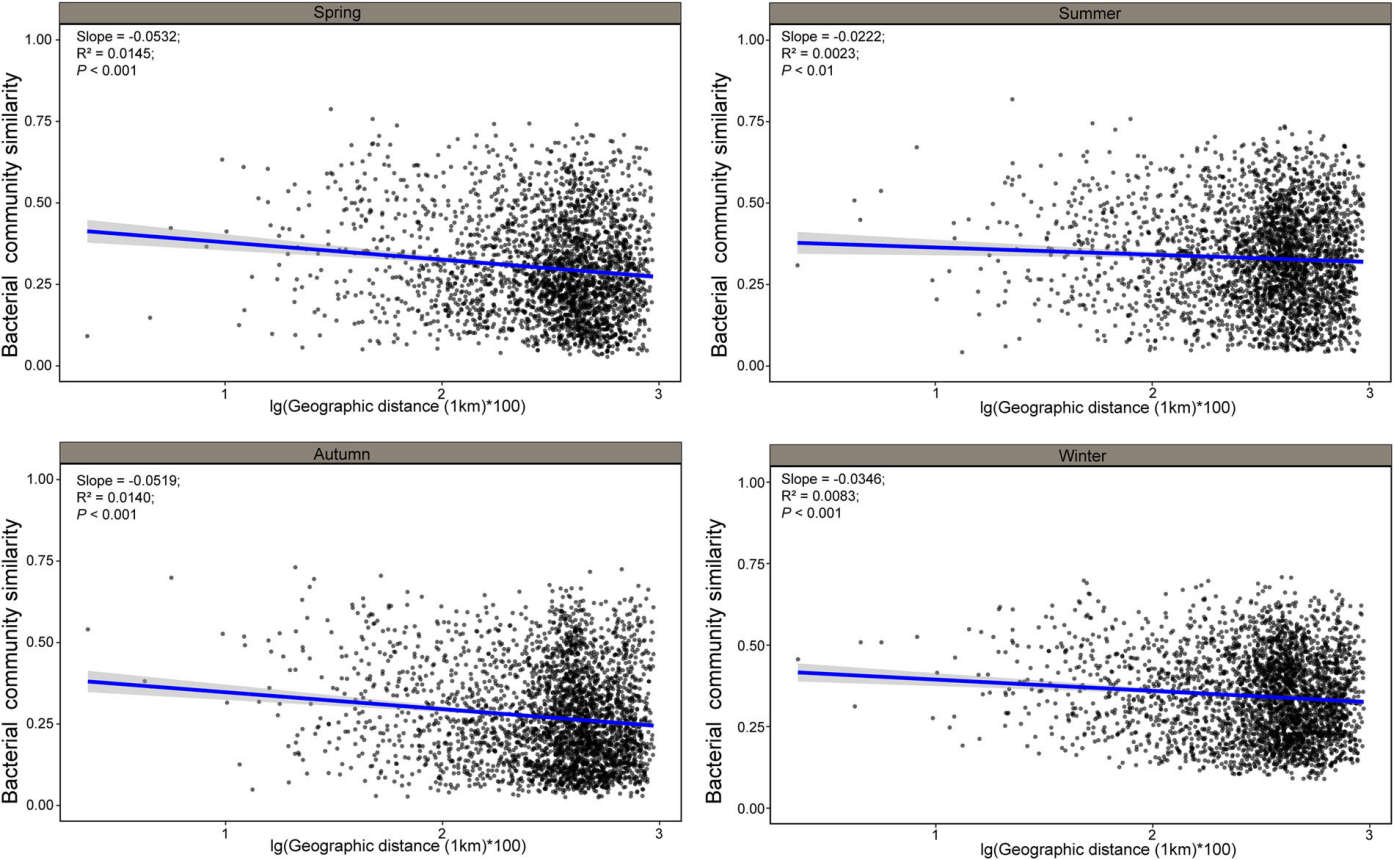
Spatial decay patterns of soil bacterial communities. Distance-decay relationships between bacterial community similarities and the geographic distances between sampling sites in (**A**) spring, (**B**) summer, (**C**) autumn and (**D**) winter. Similarity was calculated based on 1 – [dissimilarity of the Bray–Curtis distance metric]. Solid lines denote the ordinary least-squares linear regressions.

We estimated distance-decay relationships for each season for both soil ARGs and bacteria (Figs. 2, 3). Except for ARGs in the autumn (*P* = 0.18), most distance-decay patterns were significant for both ARGs and bacteria. Fitness values were remarkably low (*R*^2^ < 0.1), indicating a weak distance decay of both ARG profiles and microbial community similarities with geographic distance. These results were consistent with the nMDS visualization, which showed that ARG profiles and bacterial community composition did not clearly cluster geographically (Supplementary Fig. S5).

A NCM was used to estimate the potential dispersal limitation of ARG in soil (Supplementary Fig. S6A). There was a higher immigration rate (*m*) of ARGs in spring, indicating that the dispersal limitation of AGRs is lower in spring than the other seasons (Supplementary Fig. S6A). We further used the *NST* to quantify the role of deterministic and stochastic processes in soil ARG profiles (Supplementary Fig. S6B). *NST* values exceeded the 50% boundary point in all four seasons, suggesting that stochastic rather than deterministic process played a more important role in shaping ARG compositions.

### Multiple predictors for soil ARGs at a watershed scale

Significant co-occurrence correlations were observed in the ARG-microbial community network (Spearman’s *R*^2^ > 0.6, *P* < 0.001) (Supplementary Fig. S7). Actinobacteria, Firmicutes, and Proteobacteria were highly connected with a variety of ARG subtypes (Supplementary Fig. S7), which covered almost all major classes of antibiotics and were thought to be possible ARG hosts. For example, *lunB-02* is strongly connected with *Turicibacter* which is affiliated with Firmicutes, while *tetT* is strongly connected with *Lysobacter* which belongs to Proteobacteria. In addition, we found most single bacterial taxa to be significantly correlated with multiple ARG subtypes (Spearman’s *R*^2^ > 0.6, *P* < 0.001). Season played a role in determining the abundance of certain phyla (Supplementary Fig. S7) as annual mean temperature and annual mean precipitation were significantly correlated with Actinobacteria, Firmicutes, and Proteobacteria in soil (*P* < 0.01) (Supplementary Fig. S8).

**Fig. 4:**
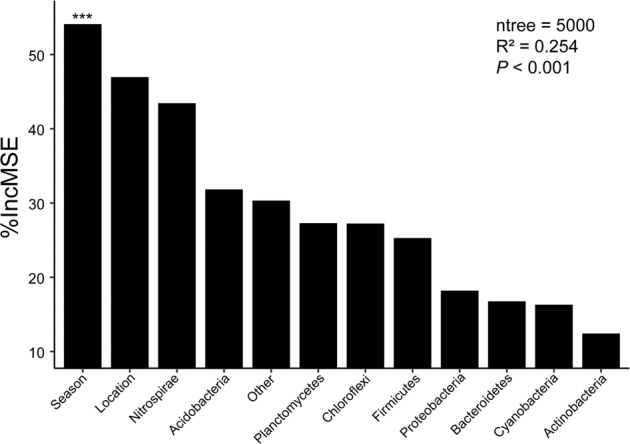
Random Forest modeling indicating the importance of different predictors on soil resistome profiles. The accuracy importance measure was computed for each tree and averaged over the forest (5000 trees). Percentage increases in the MSE (mean squared error) of variables were used to estimate the importance of these predictors, with higher MSE% values implying a greater importance of the predictors. Significance levels are as follows: ***P < 0.001. MSE mean squared error.

Finally, we used RF modeling to compare the relative importance of spatial and temporal variability and microbial community composition in shaping the distribution of ARG profiles at a watershed scale (Fig. 4). Ten of the most abundant bacterial phyla were also incorporated in this model, because the Mantel test indicated that the ARG profiles were significantly associated with changes in bacterial community composition (spearman’ *r* = 0.1698, *P* < 0.001). Our RF ranks showed that seasonal change was the most important predictor of ARG profiles (Fig. 4). Although, the importance of spatial variability in predicting of soil ARGs was weaker than temporal variability, its importance was still stronger than Nitrospirae, which was the most important microbial predictor for soil ARGs. Proteobacteria, as the most dominant phylum across time and space (Fig. 1D), exhibited a weaker predictive power regarding soil ARGs.

## Discussion

### Temporal and spatial decay relationships of soil resistomes

In the present study, a significant and negative correlation was observed between soil ARG profiles and seasonal dissimilarity (Eucliden distance), highlighting the importance of seasonal variability in predicting the soil antibiotic resistomes. It should be noted that seasonal variation is a latent variable, which is related to human activities and shifting biotic and abiotic factors (e.g., soil antibiotic concentrations). For example, prior work investigating the seasonal variation of soil antibiotics at the same site found a higher concentration of soil antibiotics in winter than summer.^42^ Consequently, antibiotic residues derived from organic fertilizers applied in winter may pose a selection pressure on the soil microbes, increasing resident antibiotic resistance and accelerating new ARG occurrence and subsequent dispersal. This was supported by our observation of a more diverse and abundant soil ARG profile in winter and spring compared to soil ARG profiles in either summer or autumn. As ARGs can be present as both extracellular DNA (eDNA) and intracellular DNA, given the persistence of eDNA in livestock waste and soil, extracellular ARG-based transformation could be playing an important role in the proliferation of ARGs in the spring and winter soil samples after the application of organic fertilizers.^43,44^ Given the importance of microbial communities in shaping soil ARG patterns^45,46^ and the prevalence of co-occurrence patterns between ARG subtypes and microbial taxa,^47,48^ one reasonable explanation of temporal ARG distribution patterns may be shifts in soil microbial communities caused by seasonal change. In addition, we found that different types of ARG displayed a different seasonal distribution pattern. For example, the abundance of genes conferring resistance to Aminoglycosides was significantly enriched in spring, while those resistant to Vancomycin were more enriched in autumn. These differences might be due to the seasonal preference of soil microbes that contain these genes. OLS analysis showed that Actinobacteria, Firmicutes and Proteobacteria, each of which contained ARGs encoding resistance to different classes of antibiotics, were significantly correlated with annual mean temperature and annual mean precipitation at a watershed scale. To give a more comprehensive understanding of soil microbial seasonal preference, further studies are needed.

Correlations between geographic distance and ARG similarity were season-dependent with significant distance-decay relationships identified in spring, summer and winter but not in autumn (*P* = 0.18). Moreover, from previous studies in the same watershed, 28–51 ARGs were detected only in farmland soils, with seasonal variation affecting farmland-related ARGs. For instance, the greatest number of farmland-related ARGs in the resistomes were detected in spring (ploughing season), while the fewest were detected in autumn (the fallow period).^45^ Based on neutral community modeling (fitness > 0.8) and *NST* index(Supplementary Fig. S6), stochastic forces rather than deterministic selection dominated soil resistomes at a watershed scale. According to Hubbell’s neutral theory (Hubbell, 2001), community similarity would be predicted to decrease with increasing spatial distance due to dispersal limitations. Thus, a distance-decay relationship would emerge even without differences in environmental conditions or niche requirements. In this study, nMDS visualization showed limited spatial differentiation in ARGs at the watershed scale. Moreover, NCM analysis estimated that the dispersal ability of most AGRs in spring was higher than other seasons, which may have partially explained the high diversity of soil ARGs in spring.

### Temporal variation driving the pattern of soil resistome

We report that seasonal variability was more important than geographical distance in shaping soil ARG profiles at a watershed scale. Previous studies have suggested that anthropogenic factors (e.g., pesticide usage) were the main forces shaping ARG patterns in cropland and sediments at a continental scale,^17,18^ while ARG composition in forest soil was regulated by the diversity of herbaceous plants and bacterial communities.^21^ However, it should be noted that both anthropogenic impacts and vegetation turnover are seasonally dependent. Furthermore, agronomic activities can also induce significant shifts in microbial community composition^49^ and in turn influence ARG patterns. Thus, season is a comprehensive proxy that encapsulates many changes to soil physiochemical conditions that may be driving soil ARG patterns.

Our results contrast with recent studies which found that spatial variability is more important than temporal variability when shaping soil microbial communities.^50,51^ These studies were conducted at either a much smaller or larger spatial scale than this study. Thus, this inconsistency is potentially due to microbial systems being studied at different spatial, temporal, and phylogenetic scales, i.e., different processes may dominate at different scales in microbial systems.^52^ Notwithstanding this, it may not be possible to completely separate the impacts of temporal and spatial variation on microbial patterns, since seasonal changes could affect microbial dispersal and environmental heterogeneity, which in turn could lead to stronger or weaker impacts of spatial variation. In the present study, significant though weak distance-decay effects were observed in soil bacterial communities. Moreover, the slopes of the distance-decay curves were steeper in the spring and autumn than those of summer and winter, indicating that seasonal variability may influence the balance between deterministic and stochastic processes in governing the assembly of soil microbial communities. Accordingly, it would be good to include more parameters that involved in seasonal change and spatial variability and quantifying the spatial and temporal variability, when designing future similar studies.

## Conclusions

To the best of our knowledge the present work evaluates, for the first time, the relative importance of spatial and temporal variability on ARG composition and abundance at a watershed scale. Our results suggested that distance-decay patterns in soil ARGs were seasonally dependent. The significant distance-decay relationships identified in spring, summer and winter had low fitness indicating weak distance-decay patterns of soil resistomes. The significant negative correlation between soil ARG profiles and seasonal dissimilarity together with RF model, indicated that seasonal variability played a major role in shaping soil ARG profiles. This study increases our understanding of soil ARGs predictors, which is crucial for predicting changes in soil ARGs due to environmental change and human disturbance.

## Supplementary Information

Supplementary data
