# Supplementary material for: Seasonal change is a major driver of soil resistomes at a watershed scale
Source: ISME Commun. 2021 May 20;1:17. doi: 10.1038/s43705-021-00018-y (PMC9723683; doi:10.1038/s43705-021-00018-y)
Supplement: Supplementary file 1 — Supplementary data [file 43705_2021_18_MOESM1_ESM.doc]

**Supplementary data for**

**Seasonal change is a major driver of soil resistomes at a watershed scale**

Qian Xiang^1,2^ Min Qiao^1,2*^ Dong Zhu^1^ Madeline Giles^3^ Roy Neilson^3^ Xiao-Ru Yang^4^ Yong-Guan Zhu^1,2,4^ Qing-Lin Chen^5*^

^1^ State Key Laboratory of Urban and Regional Ecology, Research Center for Eco-Environmental Sciences, Chinese Academy of Sciences, Beijing, 100085, China.

^2^ University of Chinese Academy of Sciences, 19A Yuquan Road, Beijing, 100049, China.

^3^ Ecological Sciences, The James Hutton Institute, Dundee, DD2 5DA, Scotland, UK.

^4^ Key Lab of Urban Environment and Health, Institute of Urban Environment, Chinese Academy of Sciences, 1799 Jimei Road, Xiamen, 361021, China.

^5^ Faculty of Veterinary and Agricultural Sciences, The University of Melbourne, Parkville, Victoria 3010, Australia.

* Corresponding Author:

[minqiao@rcees.ac.cn](mailto:minqiao@rcees.ac.cn) (Min Qiao)

[qinglin.chen@unimelb.edu.au](mailto:qinglin.chen@unimelb.edu.au) (Qing-Lin Chen)


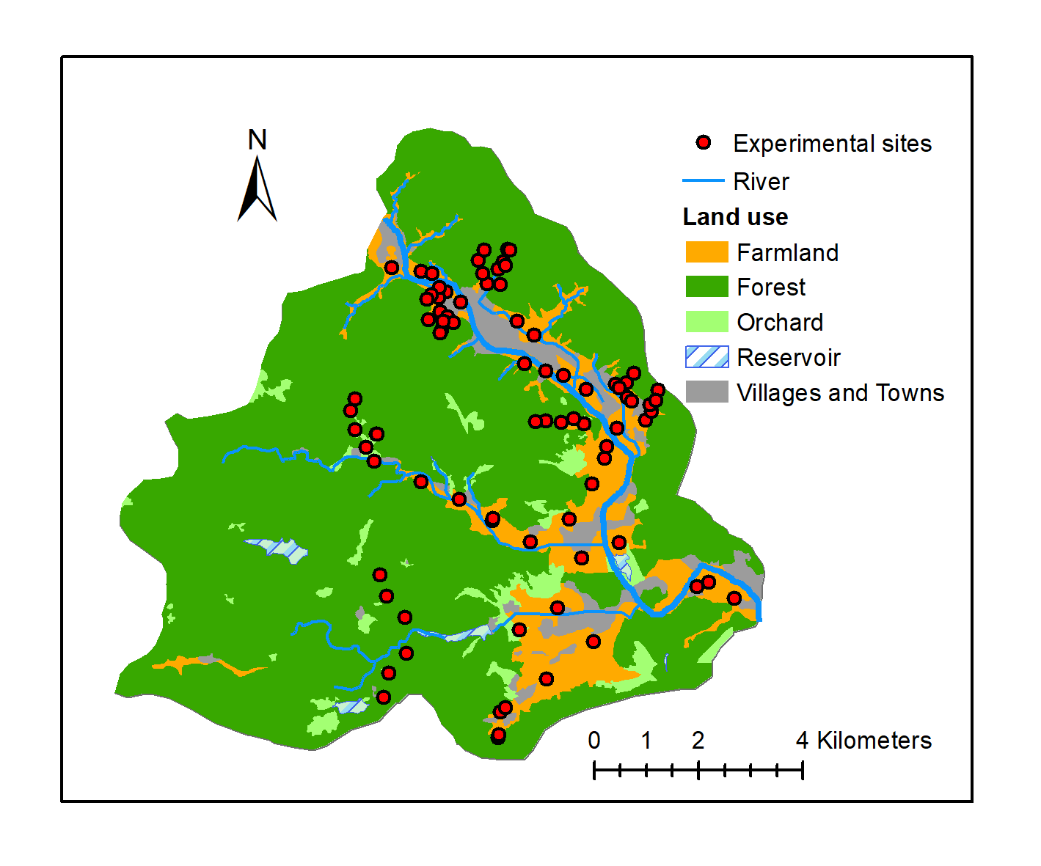


Fig. S1 Map of the sampling sites across the 89 km^2^ of the watershed.


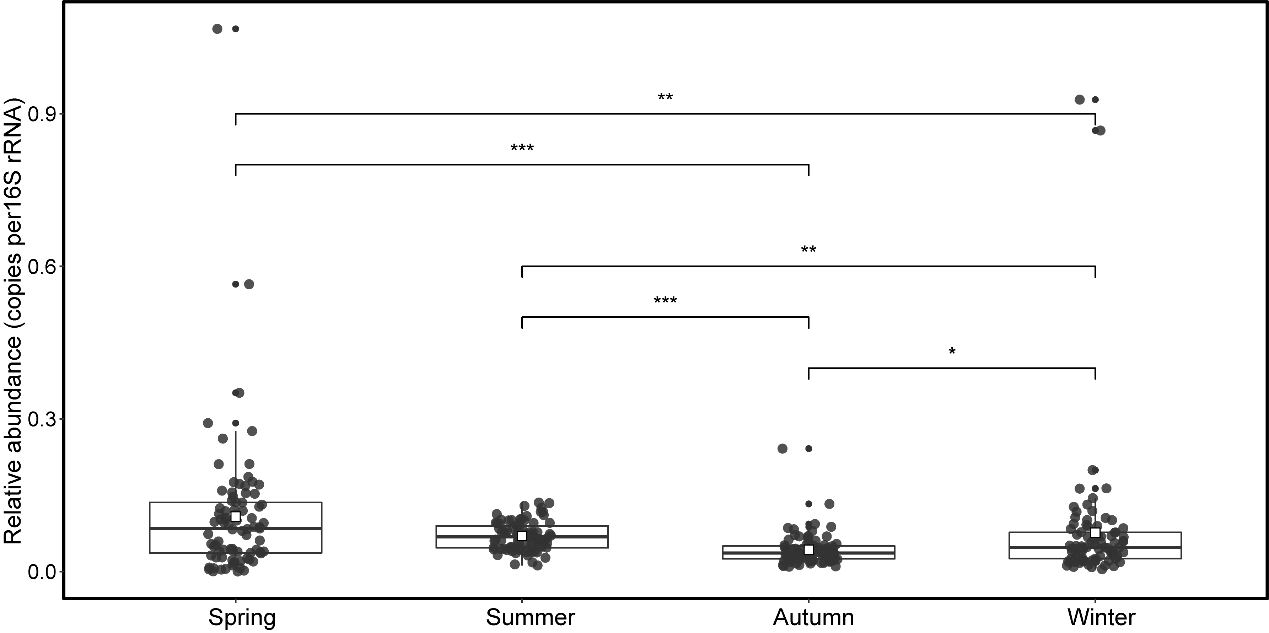


Fig. S2 Relative abundance (copies/16S rRNA gene copy) of soil ARGs in spring, summer, autumn and winter.


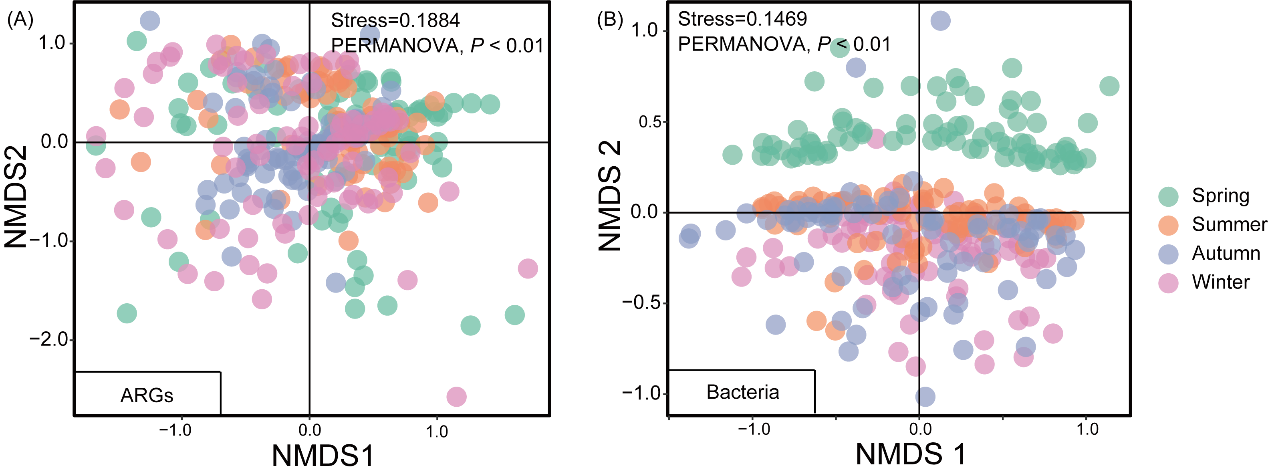


Fig. S3 Seasonal variation of the abundance of (A) ARGs and (B) bacterial communities based on Non-metric multidimensional scaling (NMDS) (PERMANOVA, *P*< 0.01, Bray-Curtis dissimilarity).


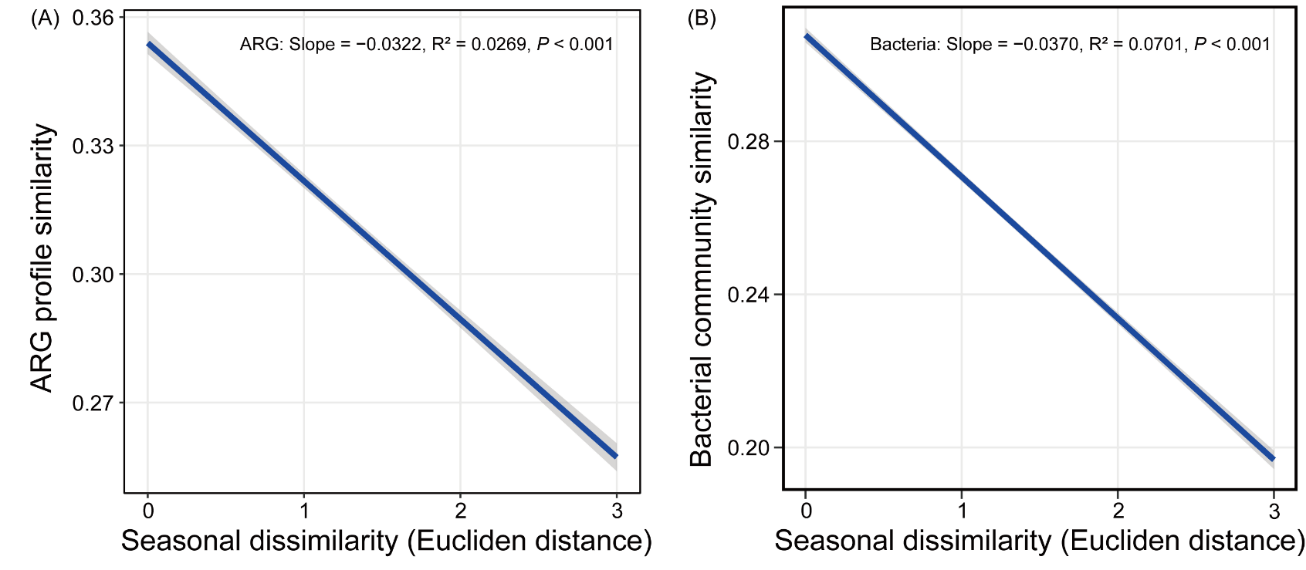


Fig. S4 Relationship between (A) soil ARG and (B) Soil bacterial communities and seasonal dissimilarity (Eucliden distance). ARG and bacterial community similarity was calculated based on 1 ̶ [dissimilarity of the Bray–Curtis distance metric]. Solid lines denote the ordinary least-squares linear regressions.


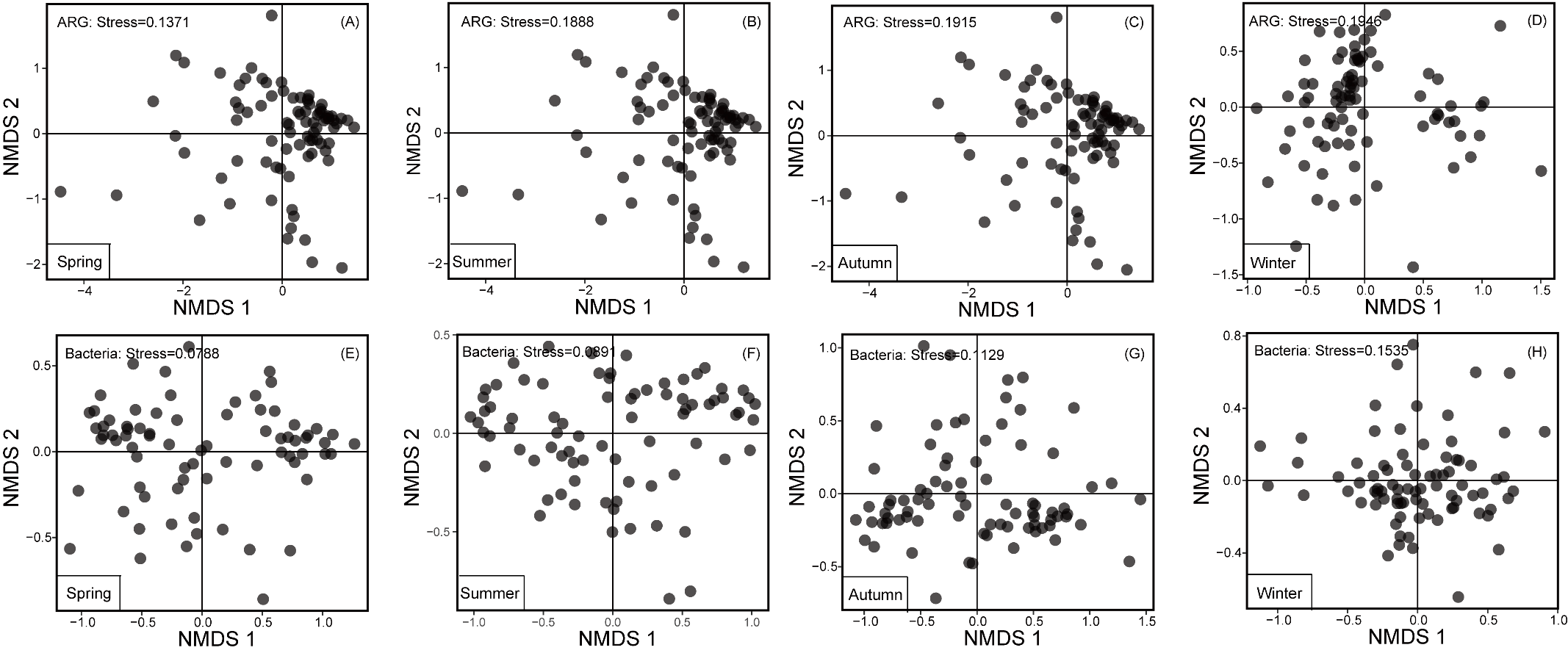


Fig. S5 Non-metric multidimensional scaling (NMDS) analysis depicting geographical distribution of antibiotic resistomes in (A) spring, (B) summer, (C) autumn, (D) winter; and bacterial communities in (E) spring, (F) summer, (G) autumn, (H) winter using Bray-Curtis distance.


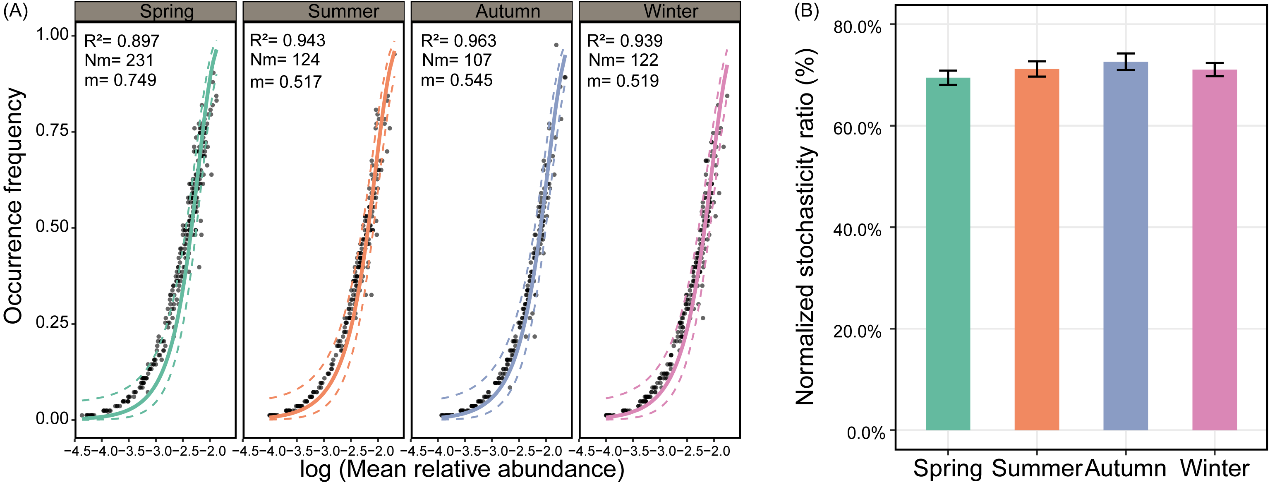


Fig. S6 (A) Fit of the neutral community model (NCM) showing the ARG predicted occurrence frequencies versus the relative abundance in soil. The solid lines indicate the best fit to the Sloan’s neutral model and the dashed lines represent 95% confidence intervals around the model prediction. R^2^ indicate the goodness of fit to the neutral model, Nm indicate the metacommunity size times immigration, and m values indicate the estimated migration rate. (B) Barplots showing the comparison of normalized stochasticity ratio (NST) between different seasons at watershed scale.


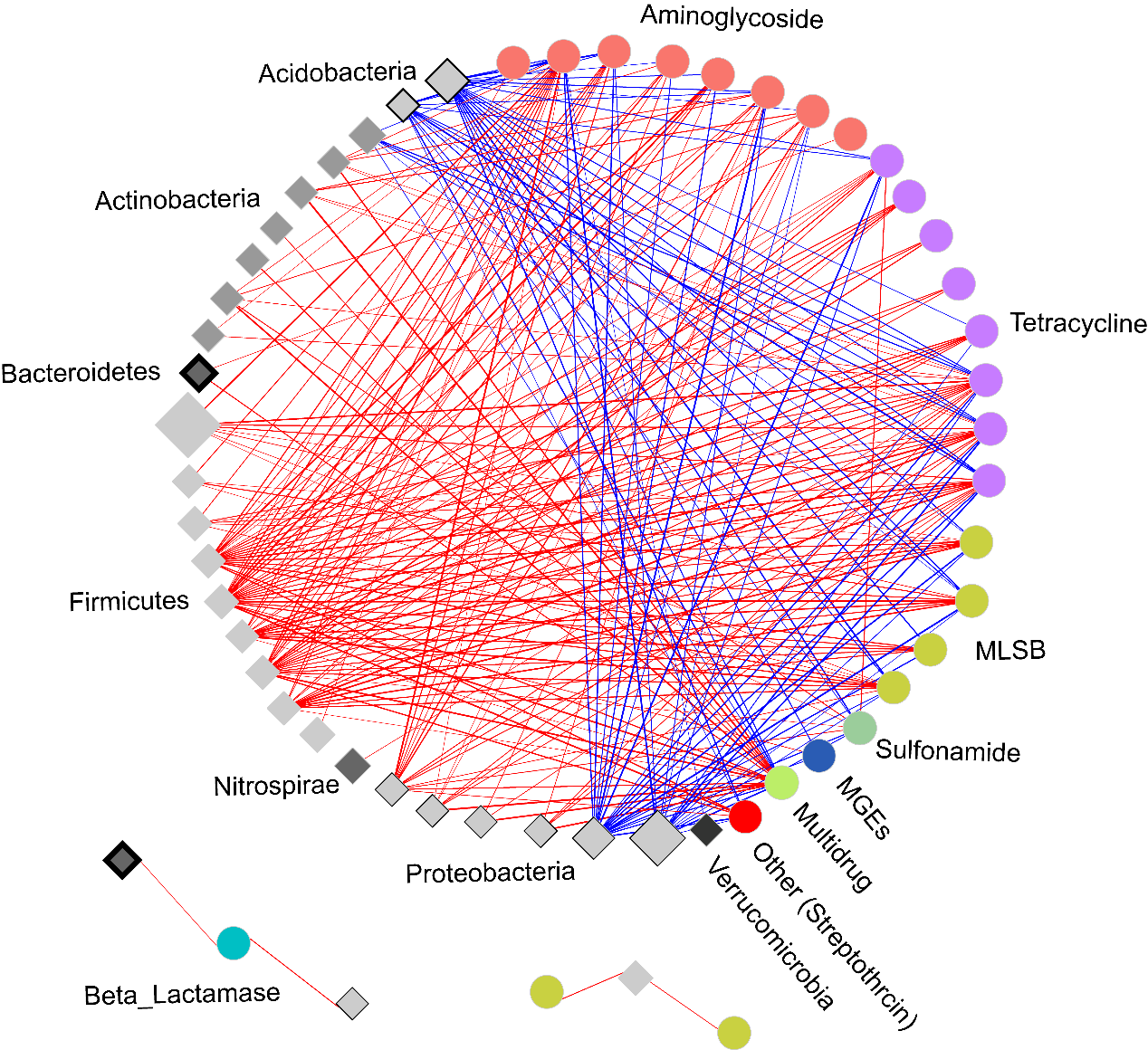


Fig. S7 The network analysis revealing the co-occurrence patterns between ARG subtypes and microbial taxa. The nodes are colored according to ARG types and microbial taxa. A connection represents a strong (Spearman's correlation coefficient R^2^ > 0.6) and significant (*P* < 0.001) correlation. Edges are weighted according to the correlation coefficient; blue means negative correlation while red means positive correlation. Node size is weighted according to the relative abundance of ARGs/microbial taxa; circles represent resistance genes and rhombuses represent bacterial taxa.


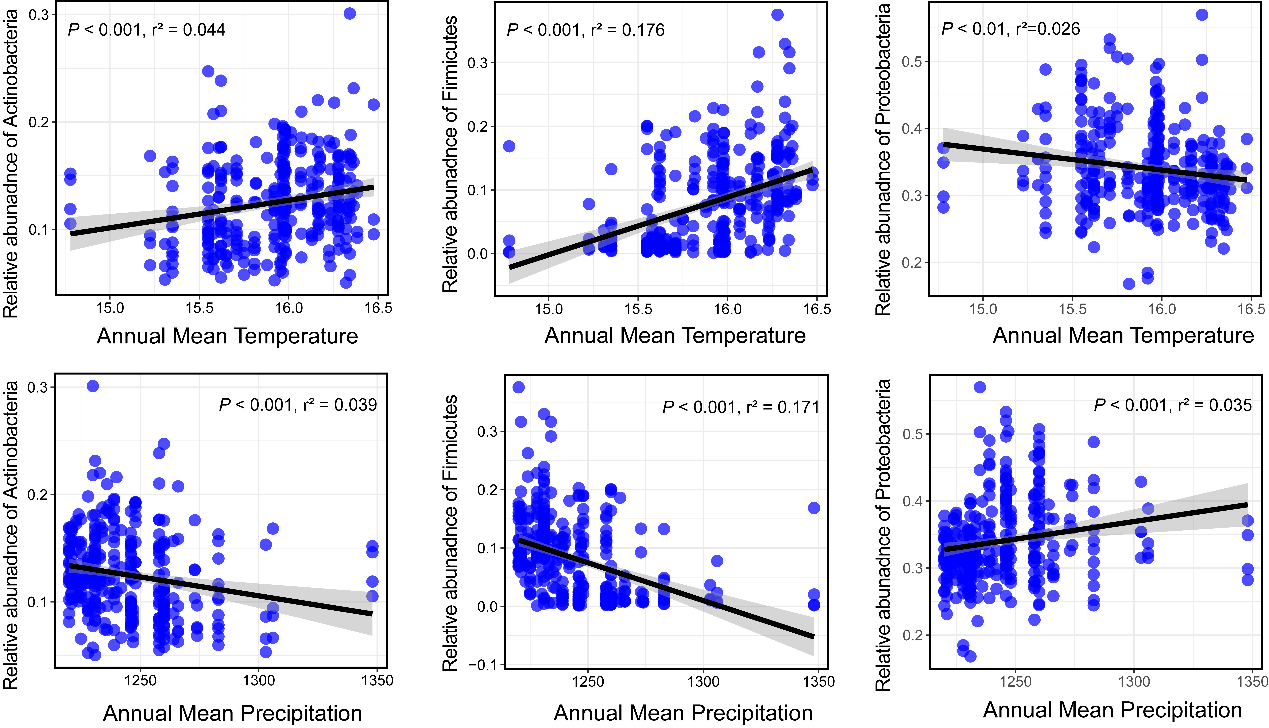


Fig. S8 Regressions between the Annual mean temperature and annual mean precipitation and Actinobacteria, Firmicutes and Proteobacteria, respectively.

**Table S1 Information of 296 genes primers**

| **Number** | **Gene Name** | **Forward Primer** | **Reverse Primer** | **Classification** |
| --- | --- | --- | --- | --- |
| 1 | 16S rRNA | GGGTTGCGCTCGTTGC | ATGGYTGTCGTCAGCTCGTG |  |
| 2 | aac | CCCTGCGTTGTGGCTATGT | TTGGCCACGCCAATCC | Aminoglycoside |
| 3 | aac(6')I1 | GACCGGATTAAGGCCGATG | CTTGCCTTGATATTCAGTTTTTATAACCA | Aminoglycoside |
| 4 | aac(6')-Ib(aka aacA4)-01 | GTTTGAGAGGCAAGGTACCGTAA | GAATGCCTGGCGTGTTTGA | Aminoglycoside |
| 5 | aac(6')-Ib(aka aacA4)-02 | CGTCGCCGAGCAACTTG | CGGTACCTTGCCTCTCAAACC | Aminoglycoside |
| 6 | aac(6')-Ib(aka aacA4)-03 | AGAAGCACGCCCGACACTT | GCTCTCCATTCAGCATTGCA | Aminoglycoside |
| 7 | aac(6')-II | CGACCCGACTCCGAACAA | GCACGAATCCTGCCTTCTCA | Aminoglycoside |
| 8 | aac(6')-Iy | GCTTTGCGGATGCCTCAAT | GGAGAACAAAAATACCTTCAAGGAAA | Aminoglycoside |
| 9 | aacA/aphD | AGAGCCTTGGGAAGATGAAGTTT | TTGATCCATACCATAGACTATCTCATCA | Aminoglycoside |
| 10 | aacC | CGTCACTTATTCGATGCCCTTAC | GTCGGGCGCGGCATA | Aminoglycoside |
| 11 | aacC1 | GGTCGTGAGTTCGGAGACGTA | GCAAGTTCCCGAGGTAATCG | Aminoglycoside |
| 12 | aacC2 | ACGGCATTCTCGATTGCTTT | CCGAGCTTCACGTAAGCATTT | Aminoglycoside |
| 13 | aacC4 | CGGCGTGGGACACGAT | AGGGAACCTTTGCCATCAACT | Aminoglycoside |
| 14 | aadA-01 | GTTGTGCACGACGACATCATT | GGCTCGAAGATACCTGCAAGAA | Aminoglycoside |
| 15 | aadA-02 | CGAGATTCTCCGCGCTGTA | GCTGCCATTCTCCAAATTGC | Aminoglycoside |
| 16 | aadA1 | AGCTAAGCGCGAACTGCAAT | TGGCTCGAAGATACCTGCAA | Aminoglycoside |
| 17 | aadA-1-01 | AAAAGCCCGAAGAGGAACTTG | CATCTTTCACAAAGATGTTGCTGTCT | Aminoglycoside |
| 18 | aadA-1-02 | CGGAATTGAAAAAACTGATCGAA | ATACCGGCTGTCCGTCATTT | Aminoglycoside |
| 19 | aadA2-01 | ACGGCTCCGCAGTGGAT | GGCCACAGTAACCAACAAATCA | Aminoglycoside |
| 20 | aadA2-02 | CTTGTCGTGCATGACGACATC | TCGAAGATACCCGCAAGAATG | Aminoglycoside |
| 21 | aadA2-03 | CAATGACATTCTTGCGGGTATC | GACCTACCAAGGCAACGCTATG | Aminoglycoside |
| 22 | aadA5-01 | ATCACGATCTTGCGATTTTGCT | CTGCGGATGGGCCTAGAAG | Aminoglycoside |
| 23 | aadA5-02 | GTTCTTGCTCTTGCTCGCATT | GATGCTCGGCAGGCAAAC | Aminoglycoside |
| 24 | aadA9-01 | CGCGGCAAGCCTATCTTG | CAAATCAGCGACCGCAGACT | Aminoglycoside |
| 25 | aadA9-02 | GGATGCACGCTTGGATGAA | CCTCTAGCGGCCGGAGTATT | Aminoglycoside |
| 26 | aadD | CCGACAACATTTCTACCATCCTT | ACCGAAGCGCTCGTCGTATA | Aminoglycoside |
| 27 | aadE | TACCTTATTGCCCTTGGAAGAGTTA | GGAACTATGTCCCTTTTAATTCTACAATCT | Aminoglycoside |
| 28 | acrA-01 | CAACGATCGGACGGGTTTC | TGGCGATGCCACCGTACT | Multidrug |
| 29 | acrA-02 | GGTCTATCACCCTACGCGCTATC | GCGCGCACGAACATACC | Multidrug |
| 30 | acrA-03 | CAGACCCGCATCGCATATT | CGACAATTTCGCGCTCATG | Multidrug |
| 31 | acrA-04 | TACTTTGCGCGCCATCTTC | CGTGCGCGAACGAACAT | Multidrug |
| 32 | acrA-05 | CGTGCGCGAACGAACA | ACTTTGCGCGCCATCTTC | Multidrug |
| 33 | acrB-01 | AGTCGGTGTTCGCCGTTAAC | CAAGGAAACGAACGCAATACC | Multidrug |
| 34 | acrF | GCGGCCAGGCACAAAA | TACGCTCTTCCCACGGTTTC | Multidrug |
| 35 | acrR-01 | GCGCTGGAGACACGACAAC | GCCTTGCTGCGAGAACAAA | Multidrug |
| 36 | acrR-02 | GATGATACCCCCTGCTGTGAGA | ACCAAACAAGAAGCGCAAGAA | Multidrug |
| 37 | adeA | CAGTTCGAGCGCCTATTTCTG | CGCCCTGACCGACCAAT | Multidrug |
| 38 | ampC/blaDHA | TGGCCGCAGCAGAAAGA | CCGTTTTATGCACCCAGGAA | Beta_Lactamase |
| 39 | ampC-01 | TGGCGTATCGGGTCAATGT | CTCCACGGGCCAGTTGAG | Beta_Lactamase |
| 40 | ampC-02 | GCAGCACGCCCCGTAA | TGTACCCATGATGCGCGTACT | Beta_Lactamase |
| 41 | ampC-04 | TCCGGTGACGCGACAGA | CAGCACGCCGGTGAAAGT | Beta_Lactamase |
| 42 | ampC-05 | CTGTTCGAGCTGGGTTCTATAAGTAAA | CAGTATCTGGTCACCGGATCGT | Beta_Lactamase |
| 43 | ampC-06 | CCGCTCAAGCTGGACCATAC | CCATATCCTGCACGTTGGTTT | Beta_Lactamase |
| 44 | ampC-09 | CAGCCGCTGATGAAAAAATATG | CAGCGAGCCCACTTCGA | Beta_Lactamase |
| 45 | aph | TTTCAGCAAGTGGATCATGTTAAAAT | CCAAGCTGTTTCCACTGTTTTTC | Aminoglycoside |
| 46 | aph(2')-Id-01 | TGAGCAGTATCATAAGTTGAGTGAAAAG | GACAGAACAATCAATCTCTATGGAATG | Aminoglycoside |
| 47 | aph(2')-Id-02 | TAAGGATATACCGACAGTTTTGGAAA | TTTAATCCCTCTTCATACCAATCCATA | Aminoglycoside |
| 48 | aph6ia | CCCATCCCATGTGTAAGGAAA | GCCACCGCTTCTGCTGTAC | Aminoglycoside |
| 49 | aphA1(aka kanR) | TGAACAAGTCTGGAAAGAAATGCA | CCTATTAATTTCCCCTCGTCAAAAA | Aminoglycoside |
| 50 | bacA-01 | CGGCTTCGTGACCTCGTT | ACAATGCGATACCAGGCAAAT | other/bacitracin |
| 51 | bacA-02 | TTCCACGACACGATTAAGTCATTG | CGGCTCTTTCGGCTTCAG | other/bacitracin |
| 52 | bla1 | GCAAGTTGAAGCGAAAGAAAAGA | TACCAGTATCAATCGCATATACACCTAA | Beta_Lactamase |
| 53 | bla-ACC-1 | CACACAGCTGATGGCTTATCTAAAA | AATAAACGCGATGGGTTCCA | Beta_Lactamase |
| 54 | blaCMY | CCGCGGCGAAATTAAGC | GCCACTGTTTGCCTGTCAGTT | Beta_Lactamase |
| 55 | blaCMY2-01 | AAAGCCTCAT GGGTGCATAAA | ATAGCTTTTGTTTGCCAGCATCA | Beta_Lactamase |
| 56 | blaCMY2-02 | GCGAGCAGCCTGAAGCA | CGGATGGGCTTGTCCTCTT | Beta_Lactamase |
| 57 | blaCTX-M-01 | GGAGGCGTGACGGCTTTT | TTCAGTGCGATCCAGACGAA | Beta_Lactamase |
| 58 | blaCTX-M-03 | CGATACCACCACGCCGTTA | GCATTGCCCAACGTCAGATT | Beta_Lactamase |
| 59 | blaCTX-M-04 | CTTGGCGTTGCGCTGAT | CGTTCATCGGCACGGTAGA | Beta_Lactamase |
| 60 | blaCTX-M-05 | GCGATAACGTGGCGATGAAT | GTCGAGACGGAACGTTTCGT | Beta_Lactamase |
| 61 | blaCTX-M-06 | CACAGTTGGTGACGTGGCTTAA | CTCCGCTGCCGGTTTTATC | Beta_Lactamase |
| 62 | blaGES | GCAATGTGCTCAACGTTCAAG | GTGCCTGAGTCAATTCTTTCAAAG | Beta_Lactamase |
| 63 | blaIMP-01 | AACACGGTTTGGTGGTTCTTGTA | GCGCTCCACAAACCAATTG | Beta_Lactamase |
| 64 | blaIMP-02 | AAGGCAGCATTTCCTCTCATTTT | GGATAGATCGAGAATTAAGCCACTCT | Beta_Lactamase |
| 65 | bla-L1 | CACCGGGTTACCAGCTGAAG | GCGAAGCTGCGCTTGTAGTC | Beta_Lactamase |
| 66 | blaMOX/blaCMY | CTATGTCAATGTGCCGAAGCA | GGCTTGTCCTCTTTCGAATAGC | Beta_Lactamase |
| 67 | blaOCH | GGCGACTTGCGCCGTAT | TTTTCTGCTCGGCCATGAG | Beta_Lactamase |
| 68 | blaOKP | GCCGCCATCACCATGAG | GGTGACGTTGTCACCGATCTG | Beta_Lactamase |
| 69 | blaOXA1/blaOXA30 | CGGATGGTTTGAAGGGTTTATTAT | TCTTGGCTTTTATGCTTGATGTTAA | Beta_Lactamase |
| 70 | blaOXA10-01 | CGCAATTATCGGCCTAGAAACT | TTGGCTTTCCGTCCCATTT | Beta_Lactamase |
| 71 | blaOXA10-02 | CGCAATTATCGGCCTAGAAACT | TTGGCTTTCCGTCCCATTT | Beta_Lactamase |
| 72 | blaOXY | CGTTCAGGCGGCAGGTT | GCCGCGATATAAGATTTGAGAATT | Beta_Lactamase |
| 73 | blaPAO | CGCCGTACAACCGGTGAT | GAAGTAATGCGGTTCTCCTTTCA | Beta_Lactamase |
| 74 | blaPER | TGCTGGTTGCTGTTTTTGTGA | CCTGCGCAATGATAGCTTCAT | Beta_Lactamase |
| 75 | blaPSE | TTGTGACCTATTCCCCTGTAATAGAA | TGCGAAGCACGCATCATC | Beta_Lactamase |
| 76 | blaROB | GCAAAGGCATGACGATTGC | CGCGCTGTTGTCGCTAAA | Beta_Lactamase |
| 77 | blaSFO | CCGCCGCCATCCAGTA | GGGCCGCCAAGATGCT | Beta_Lactamase |
| 78 | blaSHV-01 | TCCCATGATGAGCACCTTTAAA | TTCGTCACCGGCATCCA | Beta_Lactamase |
| 79 | blaSHV-02 | CTTTCCCATGATGAGCACCTTT | TCCTGCTGGCGATAGTGGAT | Beta_Lactamase |
| 80 | blaTEM | AGCATCTTACGGATGGCATGA | TCCTCCGATCGTTGTCAGAAGT | Beta_Lactamase |
| 81 | blaTLA | ACACTTTGCCATTGCTGTTTATGT | TGCAAATTTCGGCAATAATCTTT | Beta_Lactamase |
| 82 | blaVEB | CCCGATGCAAAGCGTTATG | GAAAGATTCCCTTTATCTATCTCAGACAA | Beta_Lactamase |
| 83 | blaVIM | GCACTTCTCGCGGAGATTG | CGACGGTGATGCGTACGTT | Beta_Lactamase |
| 84 | blaZ | GGAGATAAAGTAACAAATCCAGTTAGATATGA | TGCTTAATTTTCCATTTGCGATAAG | Beta_Lactamase |
| 85 | carB | GGAGTGAGGCTGACCGTAGAAG | ATCGGCGAAACGCACAAA | MLSB |
| 86 | catA1 | GGGTGAGTTTCACCAGTTTTGATT | CACCTTGTCGCCTTGCGTATA | (flor)/(chlor)/(am)phenicol |
| 87 | catB3 | GCACTCGATGCCTTCCAAAA | AGAGCCGATCCAAACGTCAT | (flor)/(chlor)/(am)phenicol |
| 88 | catB8 | CACTCGACGCCTTCCAAAG | CCGAGCCTATCCAGACATCATT | (flor)/(chlor)/(am)phenicol |
| 89 | ceoA | ATCAACACGGACCAGGACAAG | GGAAAGTCCGCTCACGATGA | Multidrug |
| 90 | cepA | AGTTGCGCAGAACAGTCCTCTT | TCGTATCTTGCCCGTCGATAAT | Beta_Lactamase |
| 91 | cfiA | GCAGCGTTGCTGGACACA | GTTCGGGATAAACGTGGTGACT | Beta_Lactamase |
| 92 | cfr | GCAAAATTCAGAGCAAGTTACGAA | AAAATGACTCCCAACCTGCTTTAT | (flor)/(chlor)/(am)phenicol |
| 93 | cfxA | TCATTCCTCGTTCAAGTTTTCAGA | TGCAGCACCAAGAGGAGATGT | Beta_Lactamase |
| 94 | cmeA | GCAGCAAAGAAGAAGCACCAA | AGCAGGGTAAGTAAAACTAAGTGGTAAATCT | Multidrug |
| 95 | cmlA1-01 | TAGGAAGCATCGGAACGTTGAT | CAGACCGAGCACGACTGTTG | (flor)/(chlor)/(am)phenicol |
| 96 | cmlA1-02 | AGGAAGCATCGGAACGTTGA | ACAGACCGAGCACGACTGTTG | (flor)/(chlor)/(am)phenicol |
| 97 | cmr | CGGCATCGTCAGTGGAATT | CGGTTCCGAAAAAGATGGAA | Multidrug |
| 98 | cmx(A) | GCGATCGCCATCCTCTGT | TCGACACGGAGCCTTGGT | (flor)/(chlor)/(am)phenicol |
| 99 | cphA-01 | GCGAGCTGCACAAGCTGAT | CGGCCCAGTCGCTCTTC | Beta_Lactamase |
| 100 | cphA-02 | GTGCTGATGGCGAGTTTCTG | GGTGTGGTAGTTGGTGTTGATCAC | Beta_Lactamase |
| 101 | dfrA1 | GGAATGGCCCTGATATTCCA | AGTCTTGCGTCCAACCAACAG | Sulfa |
| 102 | dfrA12 | CCTCTACCGAACCGTCACACA | GCGACAGCGTTGAAACAACTAC | Sulfa |
| 103 | emrD | CTCAGCAGTATGGTGGTAAGCATT | ACCAGGCGCCGAAGAAC | Multidrug |
| 104 | ereA | CCTGTGGTACGGAGAATTCATGT | ACCGCATTCGCTTTGCTT | MLSB |
| 105 | ereB | GCTTTATTTCAGGAGGCGGAAT | TTTTAAATGCCACAGCACAGAATC | MLSB |
| 106 | erm(34) | GCGCGTTGACGACGATTT | TGGTCATACTCGACGGCTAGAAC | MLSB |
| 107 | erm(35) | TTGAAAACGATGTTGCATTAAGTCA | TCTATAATCACAACTAACCACTTGAACGT | MLSB |
| 108 | erm(36) | GGCGGACCGACTTGCAT | TCTGCGTTGACGACGGTTAC | MLSB |
| 109 | ermA | TTGAGAAGGGATTTGCGAAAAG | ATATCCATCTCCACCATTAATAGTAAACC | MLSB |
| 110 | ermA/ermTR | ACATTTTACCAAGGAACTTGTGGAA | GTGGCATGACATAAACCTTCATCA | MLSB |
| 111 | ermB | TAAAGGGCATTTAACGACGAAACT | TTTATACCTCTGTTTGTTAGGGAATTGAA | MLSB |
| 112 | ermC | TTTGAAATCGGCTCAGGAAAA | ATGGTCTATTTCAATGGCAGTTACG | MLSB |
| 113 | ermF | CAGCTTTGGTTGAACATTTACGAA | AAATTCCTAAAATCACAACCGACAA | MLSB |
| 114 | ermJ/ermD | GGACTCGGCAATGGTCAGAA | CCCCGAAACGCAATATAATGTT | MLSB |
| 115 | ermK-01 | GTTTGATATTGGCATTGTCAGAGAAA | ACCATTGCCGAGTCCACTTT | MLSB |
| 116 | ermK-02 | GAGCCGCAAGCCCCTTT | GTGTTTCATTTGACGCGGAGTAA | MLSB |
| 117 | ermT-01 | GTTCACTAGCACTATTTTTAATGACAGAAGT | GAAGGGTGTCTTTTTAATACAATTAACGA | MLSB |
| 118 | ermT-02 | GTAAAATCCCTAGAGAATACTTTCATCCA | TGAGTGATATTTTTGAAGGGTGTCTT | MLSB |
| 119 | ermX | GCTCAGTGGTCCCCATGGT | ATCCCCCCGTCAACGTTT | MLSB |
| 120 | ermY | TTGTCTTTGAAAGTGAAGCAACAGT | TAACGCTAGAGAACGATTTGTATTGAG | MLSB |
| 121 | fabK | TTTCAGCTCAGCACTTTGGTCAT | AAGGCATCTTTTTCAGCCAGTTC | other |
| 122 | floR | ATTGTCTTCACGGTGTCCGTTA | CCGCGATGTCGTCGAACT | (flor)/(chlor)/(am)phenicol |
| 123 | folA | CGAGCAGTTCCTGCCAAAG | CCCAGTCATCCGGTTCATAATC | Sulfa |
| 124 | fosB | TCACTGTAACTAATGAAGCATTAGACCAT | CCATCTGGATCTGTAAAGTAAAGAGATC | other/fosfomycin |
| 125 | fosX | GATTAAGCCATATCACTTTAATTGTGAAAG | TCTCCTTCCATAATGCAAATCCA | other/fosfomycin |
| 126 | fox5 | GGTTTGCCGCTGCAGTTC | GCGGCCAGGTGACCAA | Beta_Lactamase |
| 127 | imiR | CCGGACTAGAGCTTCATGTAAGC | CCCACGCGGTACTCTTGTAAA | other |
| 128 | intI1 | GCCTTGATGTTACCCGAGAG | GATCGGTCGAATGCGTGT | Integrase |
| 129 | intI-1(clinic) | CGAACGAGTGGCGGAGGGTG | TACCCGAGAGCTTGGCACCCA | Integrase |
| 130 | intI2 | TGCTTTTCCCACCCTTACC | GACGGCTACCCTCTGTTATCTC | Integrase |
| 131 | intI3 | GCCACCACTTGTTTGAGGA | GGATGTCTGTGCCTGCTTG | Integrase |
| 132 | IS613 | AGGTTCGGACTCAATGCAACA | TTCAGCACATACCGCCTTGAT | Transposase |
| 133 | lmrA-01 | TCGACGTGACCGTAGTGAACA | CGTGACTACCCAGGTGAGTTGA | MLSB |
| 134 | lnuA-01 | TGACGCTCAACACACTCAAAAA | TTCATGCTTAAGTTCCATACGTGAA | MLSB |
| 135 | lnuB-01 | TGAACATAATCCCCTCGTTTAAAGAT | TAATTGCCCTGTTTCATCGTAAATAA | MLSB |
| 136 | lnuB-02 | AAAGGAGAAGGTGACCAATACTCTGA | GGAGCTACGTCAAACAACCAGTT | MLSB |
| 137 | lnuC | TGGTCAATATAACAGATGTAAACCAGATTT | CACCCCAGCCACCATCAA | MLSB |
| 138 | marR-01 | GCGGCGTACTGGTGAAGCTA | TGCCCTGGTCGTTGATGA | Multidrug |
| 139 | matA/mel | TAGTAGGCAAGCTCGGTGTTGA | CCTGTGCTATTTTAAGCCTTGTTTCT | MLSB |
| 140 | mdetl1 | ATACAGCAGTGGATATTGGTTTAATTGT | TGCATAAGGTGAATGTTCCATGA | Multidrug |
| 141 | mdtA | CCTAACGGGCGTGACTTCA | TTCACCTGTTTCAAGGGTCAAA | MLSB |
| 142 | mdtE/yhiU | CGTCGGCGCACTCGTT | TCCAGACGTTGTACGGTAACCA | Multidrug |
| 143 | mecA | GGTTACGGACAAGGTGAAATACTGAT | TGTCTTTTAATAAGTGAGGTGCGTTAATA | Beta_Lactamase |
| 144 | mefA | CCGTAGCATTGGAACAGCTTTT | AAACGGAGTATAAGAGTGCTGCAA | MLSB |
| 145 | mepA | ATCGGTCGCTCTTCGTTCAC | ATAAATAGGATCGAGCTGCTGGAT | Multidrug |
| 146 | mexA | AGGACAACGCTATGCAACGAA | CCGGAAAGGGCCGAAAT | Multidrug |
| 147 | mexD | TTGCCACTGGCTTTCATGAG | CACTGCGGAGAACTGTCTGTAGA | Multidrug |
| 148 | mexE | GGTCAGCACCGACAAGGTCTAC | AGCTCGACGTACTTGAGGAACAC | Multidrug |
| 149 | mexF | CCGCGAGAAGGCCAAGA | TTGAGTTCGGCGGTGATGA | Multidrug |
| 150 | mphA-01 | CTGACGCGCTCCGTGTT | GGTGGTGCATGGCGATCT | MLSB |
| 151 | mphA-02 | TGATGACCCTGCCATCGA | TTCGCGAGCCCCTCTTC | MLSB |
| 152 | mphB | CGCAGCGCTTGATCTTGTAG | TTACTGCATCCATACGCTGCTT | MLSB |
| 153 | mphC | CGTTTGAAGTACCGAATTGGAAA | GCTGCGGGTTTGCCTGTA | MLSB |
| 154 | msrA-01 | CTGCTAACACAAGTACGATTCCAAAT | TCAAGTAAAGTTGTCTTACCTACACCATT | MLSB |
| 155 | msrC-01 | TCAGACCGGATCGGTTGTC | CCTATTTTTTGGAGTCTTCTCTCTAATGTT | MLSB |
| 156 | mtrC-01 | GGACGGGAAGATGGTCCAA | CGTAGCGTTCCGGTTCGAT | Multidrug |
| 157 | mtrC-02 | CGGAGTCCATCGACCATTTG | ATCGTCGGCAAGGAGAATCA | Multidrug |
| 158 | mtrD-02 | GGTCGGCACGCTCTTGTC | TGAAGAATTTGCGCACCACTAC | Multidrug |
| 159 | mtrD-03 | CCGCCAAGCCGATATAGACA | GGCCGGGTTGCCAAA | Multidrug |
| 160 | ndm-1 | ATTAGCCGCTGCATTGAT | CATGTCGAGATAGGAAGTG | Beta_Lactamase |
| 161 | nimE | TGCGCCAAGATAGGGCATA | GTCGTGAATTCGGCAGGTTTA | other/nitroimidazole |
| 162 | nisB | GGGAGAGTTGCCGATGTTGTA | AGCCACTCGTTAAAGGGCAAT | other |
| 163 | oleC | CCCGGAGTCGATGTTCGA | GCCGAAGACGTACACGAACAG | MLSB |
| 164 | oprD | ATGAAGTGGAGCGCCATTG | GGCCACGGCGAACTGA | Multidrug |
| 165 | oprJ | ACGAGAGTGGCGTCGACAA | AAGGCGATCTCGTTGAGGAA | Multidrug |
| 166 | pbp | CCGGTGCCATTGGTTTAGA | AAAATAGCCGCCCCAAGATT | Beta_Lactamase |
| 167 | pbp2x | TTTCATAAGTATCTGGACATGGAAGAA | CCAAAGGAAACTTGCTTGAGATTAG | Beta_Lactamase |
| 168 | Pbp5 | GGCGAACTTCTAATTAATCCTATCCA | CGCCGATGACATTCTTCTTATCTT | Beta_Lactamase |
| 169 | penA | AGACGGTAACGTATAACTTTTTGAAAGA | GCGTGTAGCCGGCAATG | Beta_Lactamase |
| 170 | pikR1 | TCGACATGCGTGACGAGATT | CCGCGAATTAGGCCAGAA | MLSB |
| 171 | pikR2 | TCGTGGGCCAGGTGAAGA | TTCCCCTTGCCGGTGAA | MLSB |
| 172 | pmrA | TTTGCAGGTTTTGTTCCTAATGC | GCAGAGCCTGATTTCTCCTTTG | Multidrug |
| 173 | pncA | GCAATCGAGGCGGTGTTC | TTGCCGCAGCCAATTCA | other/Pyrazinamide |
| 174 | putitive multidrug | AATTTTGCCGATTATTGCTGAAA | GATTGTCATCATTCGTTTATCACCAA | Multidrug |
| 175 | qac | CAATAATAACCGAAATAATAGGGACAAGTT | AATAAGTGTTCCTAGTGTTGGCCATAG | Multidrug |
| 176 | qacA | TGGCAATAGGAGCTATGGTGTTT | AAGGTAACACTATTTTCGGTCCAAATC | Multidrug |
| 177 | qacA/qacB | TTTAGGCAGCCTCGCTTCA | CCGAATCCAAATAAAACCCAATAA | Multidrug |
| 178 | qacEdelta1-01 | TCGCAACATCCGCATTAAAA | ATGGATTTCAGAACCAGAGAAAGAAA | Multidrug |
| 179 | qacEdelta1-02 | CCCCTTCCGCCGTTGT | CGACCAGACTGCATAAGCAACA | Multidrug |
| 180 | qacH-01 | GTGGCAGCTATCGCTTGGAT | CCAACGAACGCCCACAA | Multidrug |
| 181 | qacH-02 | CATCGTGCTTGTGGCAGCTA | TGAACGCCCAGAAGTCTAGTTTT | Multidrug |
| 182 | qnrA | AGGATTTCTCACGCCAGGATT | CCGCTTTCAATGAAACTGCAA | (flor)/(chlor)/(am)phenicol |
| 183 | rarD-02 | TGACGCATCGCGTGATCT | AAATTTTCTGTGGCGTCTGAATC | Multidrug |
| 184 | sat4 | GAATGGGCAAAGCATAAAAACTTG | CCGATTTTGAAACCACAATTATGATA | other/streptothricin |
| 185 | sdeB | CACTACCGCTTCCGCACTTAA | TGAAAAAACGGGAAAAGTCCAT | Multidrug |
| 186 | spcN-01 | AAAAGTTCGATGAAACACGCCTAT | TCCAGTGGTAGTCCCCGAATC | Aminoglycoside |
| 187 | spcN-02 | CAGAATCTTCCTGAAAAGTTTGATGAA | CGCAGACACGCCGAATC | Aminoglycoside |
| 188 | speA | GCAAGAGGTATTTGCTCAACAAGA | CAGGGTCACCCTCATAAAGAAAA | other |
| 189 | str | AATGAGTTTTGGAGTGTCTCAACGTA | AATCAAAACCCCTATTAAAGCCAAT | Aminoglycoside |
| 190 | strA | CCGGTGGCATTTGAGAAAAA | GTGGCTCAACCTGCGAAAAG | Aminoglycoside |
| 191 | strB | GCTCGGTCGTGAGAACAATCT | CAATTTCGGTCGCCTGGTAGT | Aminoglycoside |
| 192 | sul1 | CAGCGCTATGCGCTCAAG | ATCCCGCTGCGCTGAGT | Sulfa |
| 193 | sul2 | TCATCTGCCAAACTCGTCGTTA | GTCAAAGAACGCCGCAATGT | sulfa |
| 194 | sulA/folP-01 | CAGGCTCGTAAATTGATAGCAGAAG | CTTTCCTTGCGAATCGCTTT | Sulfa |
| 195 | sulA/folP-03 | CACGGCTTCGGCTCATGT | TGCCATCCTGTGACTAGCTACGT | Sulfa |
| 196 | tet(32) | CCATTACTTCGGACAACGGTAGA | CAATCTCTGTGAGGGCATTTAACA | Tet_Resistance |
| 197 | tet(34) | CTTAGCGCAAACAGCAATCAGT | CGGTGATACAGCGCGTAAACT | Tet_Resistance |
| 198 | tet(35) | ACCCCATGACGTACCTGTAGAGA | CAACCCACACTGGCTACCAGTT | Tet_Resistance |
| 199 | tet(36)-01 | AGAATACTCAGCAGAGGTCAGTTCCT | TGGTAGGTCGATAACCCGAAAAT | Tet_Resistance |
| 200 | tet(36)-02 | TGCAGGAAAGACCTCCATTACAG | CTTTGTCCACACTTCCACGTACTATG | Tet_Resistance |
| 201 | tet(37) | GAGAACGTTGAAAAGGTGGTGAA | AACCAAGCCTGGATCAGTCTCA | Tet_Resistance |
| 202 | tetA-01 | GCTGTTTGTTCTGCCGGAAA | GGTTAAGTTCCTTGAACGCAAACT | Tet_Resistance |
| 203 | tetA-02 | CTCACCAGCCTGACCTCGAT | CACGTTGTTATAGAAGCCGCATAG | Tet_Resistance |
| 204 | tetB-01 | AGTGCGCTTTGGATGCTGTA | AGCCCCAGTAGCTCCTGTGA | Tet_Resistance |
| 205 | tetB-02 | GCCCAGTGCTGTTGTTGTCAT | TGAAAGCAAACGGCCTAAATACA | Tet_Resistance |
| 206 | tetC-01 | CATATCGCAATACATGCGAAAAA | AAAGCCGCGGTAAATAGCAA | Tet_Resistance |
| 207 | tetC-02 | ACTGGTAAGGTAAACGCCATTGTC | ATGCATAAACCAGCCATTGAGTAAG | Tet_Resistance |
| 208 | tetD-01 | TGCCGCGTTTGATTACACA | CACCAGTGATCCCGGAGATAA | Tet_Resistance |
| 209 | tetD-02 | TGTCATCGCGCTGGTGATT | CATCCGCTTCCGGGAGAT | Tet_Resistance |
| 210 | tetE | TTGGCGCTGTATGCAATGAT | CGACGACCTATGCGATCTGA | Tet_Resistance |
| 211 | tetG-01 | TCAACCATTGCCGATTCGA | TGGCCCGGCAATCATG | Tet_Resistance |
| 212 | tetG-02 | CATCAGCGCCGGTCTTATG | CCCCATGTAGCCGAACCA | Tet_Resistance |
| 213 | tetH | TTTGGGTCATCTTACCAGCATTAA | TTGCGCATTATCATCGACAGA | Tet_Resistance |
| 214 | tetJ | GGGTGCCGCATTAGATTACCT | TCGTCCAATGTAGAGCATCCATA | Tet_Resistance |
| 215 | tetK | CAGCAGTCATTGGAAAATTATCTGATTATA | CCTTGTACTAACCTACCAAAAATCAAAATA | Tet_Resistance |
| 216 | tetL-01 | AGCCCGATTTATTCAAGGAATTG | CAAATGCTTTCCCCCTGTTCT | Tet_Resistance |
| 217 | tetL-02 | ATGGTTGTAGTTGCGCGCTATAT | ATCGCTGGACCGACTCCTT | Tet_Resistance |
| 218 | tetM-01 | CATCATAGACACGCCAGGACATAT | CGCCATCTTTTGCAGAAATCA | Tet_Resistance |
| 219 | tetM-02 | TAATATTGGAGTTTTAGCTCATGTTGATG | CCTCTCTGACGTTCTAAAAGCGTATTAT | Tet_Resistance |
| 220 | tetO-01 | ATGTGGATACTACAACGCATGAGATT | TGCCTCCACATGATATTTTTCCT | Tet_Resistance |
| 221 | tetPA | AGTTGCAGATGTGTATAGTCGTAAACTATCTATT | TGCTACAAGTACGAAAACAAAACTAGAA | Tet_Resistance |
| 222 | tetPB-01 | ACACCTGGACACGCTGATTTT | ACCGTCTAGAACGCGGAATG | Tet_Resistance |
| 223 | tetPB-02 | TGATACACCTGGACACGCTGAT | CGTCCAAAACGCGGAATG | Tet_Resistance |
| 224 | tetPB-03 | TGGGCGACAGTAGGCTTAGAA | TGACCCTACTGAAACATTAGAAATATACCT | Tet_Resistance |
| 225 | tetPB-04 | AGTGGTGCAAATACTGAAAAAGTTGT | TTTGTTCCTTCGTTTTGGACAGA | Tet_Resistance |
| 226 | tetPB-05 | CTGAAGTGGAGCGATCATTCC | CCCTCAACGGCAGAAATAACTAA | Tet_Resistance |
| 227 | tetQ | CGCCTCAGAAGTAAGTTCATACACTAAG | TCGTTCATGCGGATATTATCAGAAT | Tet_Resistance |
| 228 | tetR-02 | CGCGATAGACGCCTTCGA | TCCTGACAACGAGCCTCCTT | Tet_Resistance |
| 229 | tetR-03 | CGCGATGGAGCAAAAGTACAT | AGTGAAAAACCTTGTTGGCATAAAA | Tet_Resistance |
| 230 | tetS | TTAAGGACAAACTTTCTGACGACATC | TGTCTCCCATTGTTCTGGTTCA | Tet_Resistance |
| 231 | tetT | CCATATAGAGGTTCCACCAAATCC | TGACCCTATTGGTAGTGGTTCTATTG | Tet_Resistance |
| 232 | tetU-01 | GTGGCAAAGCAACGGATTG | TGCGGGCTTGCAAAACTATC | Tet_Resistance |
| 233 | tetV | GCGGGAACGACGATGTATATC | CCGCTATCTCACGACCATGAT | Tet_Resistance |
| 234 | tetX | AAATTTGTTACCGACACGGAAGTT | CATAGCTGAAAAAATCCAGGACAGTT | Tet_Resistance |
| 235 | tnpA-01 | CATCATCGGACGGACAGAATT | GTCGGAGATGTGGGTGTAGAAAGT | Transposase |
| 236 | tnpA-02 | GGGCGGGTCGATTGAAA | GTGGGCGGGATCTGCTT | Transposase |
| 237 | tnpA-03 | AATTGATGCGGACGGCTTAA | TCACCAAACTGTTTATGGAGTCGTT | Transposase |
| 238 | tnpA-04 | CCGATCACGGAAAGCTCAAG | GGCTCGCATGACTTCGAATC | Transposase |
| 239 | tnpA-05 | GCCGCACTGTCGATTTTTATC | GCGGGATCTGCCACTTCTT | Transposase |
| 240 | tnpA-07 | GAAACCGATGCTACAATATCCAATTT | CAGCACCGTTTGCAGTGTAAG | Transposase |
| 241 | tolC-01 | GGCCGAGAACCTGATGCA | AGACTTACGCAATTCCGGGTTA | Multidrug |
| 242 | tolC-02 | CAGGCAGAGAACCTGATGCA | CGCAATTCCGGGTTGCT | Multidrug |
| 243 | tolC-03 | GCCAGGCAGAGAACCTGATG | CGCAATTCCGGGTTGCT | Multidrug |
| 244 | Tp614 | GGAAATCAACGGCATCCAGTT | CATCCATGCGCTTTTGTCTCT | Transposase |
| 245 | ttgA | ACGCCAATGCCAAACGATT | GTCACGGCGCAGCTTGA | Multidrug |
| 246 | ttgB | TCGCCCTGGATGTACACCTT | ACCATTGCCGACATCAACAAC | Multidrug |
| 247 | vanA | AAAAGGCTCTGAAAACGCAGTTAT | CGGCCGTTATCTTGTAAAAACAT | Vancomycin |
| 248 | vanB-01 | TTGTCGGCGAAGTGGATCA | AGCCTTTTTCCGGCTCGTT | Vancomycin |
| 249 | vanB-02 | CCGGTCGAGGAACGAAATC | TCCTCCTGCAAAAAAAGATCAAC | Vancomycin |
| 250 | vanC-01 | ACAGGGATTGGCTATGAACCAT | TGACTGGCGATGATTTGACTATG | Vancomycin |
| 251 | vanC-03 | AAATCAATACTATGCCGGGCTTT | CCGACCGCTGCCATCA | Vancomycin |
| 252 | vanC1 | AGGCGATAGCGGGTATTGAA | CAATCGTCAATTGCTCATTTCC | Vancomycin |
| 253 | vanC2/vanC3 | TTTGACTGTCGGTGCTTGTGA | TCAATCGTTTCAGGCAATGG | Vancomycin |
| 254 | vanG | ATTTGAATTGGCAGGTATACAGGTTA | TGATTTGTCTTTGTCCATACATAATGC | Vancomycin |
| 255 | vanHB | GAGGTTTCCGAGGCGACAA | CTCTCGGCGGCAGTCGTAT | Vancomycin |
| 256 | vanHD | GTGGCCGATTATACCGTCATG | CGCAGGTCATTCAGGCAAT | Vancomycin |
| 257 | vanRA-01 | CCCTTACTCCCACCGAGTTTT | TTCGTCGCCCCATATCTCAT | Vancomycin |
| 258 | vanRA-02 | CCACTCCGGCCTTGTCATT | GCTAACCACATTCCCCTTGTTTT | Vancomycin |
| 259 | vanRB | GCCCTGTCGGATGACGAA | TTACATAGTCGTCTGCCTCTGCAT | Vancomycin |
| 260 | vanRC | TGCGGGAAAAACTGAACGA | CCCCCCATACGGTTTTGATTA | Vancomycin |
| 261 | vanRC4 | AGTGCTTTGGCTTATCTCGAAAA | TCCGGCAGCATCACATCTAA | Vancomycin |
| 262 | vanRD | TTATAATGGCAAGGATGCACTAAAGT | CGTCTACATCCGGAAGCATGA | Vancomycin |
| 263 | vanSA | CGCGTCATGCTTTCAAAATTC | TCCGCAGAAAGCTCAATTTGTT | Vancomycin |
| 264 | vanSB | GCGCGGCAAATGACAAC | TTTGCCATTTTATTCGCACTGT | Vancomycin |
| 265 | vanSC-02 | GCCATCAGCGAGTCTGATGA | CAGCTGGGATCGTTTTTCCTT | Vancomycin |
| 266 | vanSE | TGGCCGAAGAAGCAGGAA | CAATAATACTCGTCAAAGGAGTTCTCA | Vancomycin |
| 267 | vanTC-01 | CACACGCATTTTTTCCCATCTAG | CAGCCAACAGATCATCAAAACAA | Vancomycin |
| 268 | vanTC-02 | ACAGTTGCCGCTGGTGAAG | CGTGGCTGGTCGATCAAAA | Vancomycin |
| 269 | vanTE | GTGGTGCCAAGGAAGTTGCT | CGTAGCCACCGCAAAAAAAT | Vancomycin |
| 270 | vanTG | CGTGTAGCCGTTCCGTTCTT | CGGCATTACAGGTATATCTGGAAA | Vancomycin |
| 271 | vanWB | CGGACAAAGATACCCCCTATAAAG | AAATAGTAAATTGCTCATCTGGCACAT | Vancomycin |
| 272 | vanWG | ACATTTTCATTTTGGCAGCTTGTAC | CCGCCATAAGAGCCTACAATCT | Vancomycin |
| 273 | vanXA | CGCTAAATATGCCACTTGGGATA | TCAAAAGCGATTCAGCCAACT | Vancomycin |
| 274 | vanXB | AGGCACAAAATCGAAGATGCTT | GGGTATGGCTCATCAATCAACTT | Vancomycin |
| 275 | vanXD | TAAACCGTGTTATGGGAACGAA | GCGATAGCCGTCCCATAAGA | Vancomycin |
| 276 | vanYB | GGCTAAAGCGGAAGCAGAAA | GATATCCACAGCAAGACCAAGCT | Vancomycin |
| 277 | vanYD-01 | AAGGCGATACCCTGACTGTCA | ATTGCCGGACGGAAGCA | Vancomycin |
| 278 | vanYD-02 | CAAACGGAAGAGAGGTCACTTACA | CGGACGGTAATAGGGACTGTTC | Vancomycin |
| 279 | vatB-01 | GGAAAAAGCAACTCCATCTCTTGA | TCCTGGCATAACAGTAACATTCTGA | MLSB |
| 280 | vatB-02 | TTGGGAAAAAGCAACTCCATCT | CAATCCACACATCATTTCCAACA | MLSB |
| 281 | vatC-01 | CGGAAATTGGGAACGATGTT | GCAATAATAGCCCCGTTTCCTA | MLSB |
| 282 | vatC-02 | CGATGTTTGGATTGGACGAGAT | GCTGCAATAATAGCCCCGTTT | MLSB |
| 283 | vatE-01 | GGTGCCATTATCGGAGCAAAT | TTGGATTGCCACCGACAAT | MLSB |
| 284 | vatE-02 | GACCGTCCTACCAGGCGTAA | TTGGATTGCCACCGACAATT | MLSB |
| 285 | vgaA-01 | CGAGTATTGTGGAAAGCAGCTAGTT | CCCGTACCGTTAGAGCCGATA | MLSB |
| 286 | vgaA-02 | GACGGGTATTGTGGAAAGCAA | TTTCCTGTACCATTAGATCCGATAATT | MLSB |
| 287 | vgb-01 | AGGGAGGGTATCCATGCAGAT | ACCAAATGCGCCCGTTT | MLSB |
| 288 | vgbB-01 | CAGCCGGATTCTGGTCCTT | TACGATCTCCATTCAATTGGGTAAA | MLSB |
| 289 | vgbB-02 | ATACGAGCTGCCTAATAAAGGATCTT | TGTGAACCACAGGGCATTATCA | MLSB |
| 290 | yceE/mdtG-01 | TGGCACAAAATATCTGGCAGTT | TTGTGTGGCGATAAGAGCATTAG | Multidrug |
| 291 | yceE/mdtG-02 | TTATCTGTTTTCTGCTCACCTTCTTTT | GCGTGGTGACAAACAGGCTTA | Multidrug |
| 292 | yceL/mdtH-01 | TCGGGATGGTGGGCAAT | CGATAACCGAGCCGATGTAGA | Multidrug |
| 293 | yceL/mdtH-02 | CGCGTGAAACCTTAAGTGCTT | AGACGGCTAAACCCCATATAGCT | Multidrug |
| 294 | yceL/mdtH-03 | CTGCCGTTAAATGGATGTATGC | ACTCCAGCGGGCGATAGG | Multidrug |
| 295 | yidY/mdtL-01 | GCAGTTGCATATCGCCTTCTC | CTTCCCGGCAAACAGCAT | Multidrug |
| 296 | yidY/mdtL-02 | TGCTGATCGGGATTCTGATTG | CAGGCGCGACGAACATAAT | Multidrug |

**Table S2 Information of sampling sites and annual mean temperature and precipitation.**

| **Samples** | **Longitude** | **Latitude** | **Annual Mean Temperature** | **Annual Precipitation** |
| --- | --- | --- | --- | --- |
| Site 1 | 121.2792 | 29.8292 | 15.9208 | 1228 |
| Site 2 | 121.2842 | 29.8256 | 15.7083 | 1246 |
| Site 3 | 121.2812 | 29.8251 | 15.9208 | 1228 |
| Site 4 | 121.2804 | 29.8243 | 15.6208 | 1258 |
| Site 5 | 121.2827 | 29.8247 | 15.6208 | 1258 |
| Site 6 | 121.2830 | 29.8223 | 15.6208 | 1258 |
| Site 7 | 121.2808 | 29.8208 | 15.6208 | 1258 |
| Site 8 | 121.2845 | 29.8213 | 15.9750 | 1231 |
| Site 9 | 121.2836 | 29.8206 | 15.9750 | 1231 |
| Site 10 | 121.2831 | 29.8185 | 15.6208 | 1258 |
| Site 11 | 121.2922 | 29.8271 | 15.5500 | 1260 |
| Site 12 | 121.2914 | 29.8289 | 15.7083 | 1246 |
| Site 13 | 121.2905 | 29.8312 | 15.7083 | 1246 |
| Site 14 | 121.2917 | 29.8330 | 15.7083 | 1246 |
| Site 15 | 121.2954 | 29.8310 | 15.5500 | 1260 |
| Site 16 | 121.2963 | 29.8332 | 15.5500 | 1260 |
| Site 17 | 121.2967 | 29.8331 | 15.5500 | 1260 |
| Site 18 | 121.2945 | 29.8297 | 15.5500 | 1260 |
| Site 19 | 121.2959 | 29.8304 | 15.5500 | 1260 |
| Site 20 | 121.2949 | 29.8271 | 15.5500 | 1260 |
| Site 21 | 121.2984 | 29.8207 | 16.0667 | 1225 |
| Site 22 | 121.3017 | 29.8184 | 16.0667 | 1223 |
| Site 23 | 121.3000 | 29.8135 | 16.0125 | 1237 |
| Site 24 | 121.3041 | 29.8122 | 16.1667 | 1224 |
| Site 25 | 121.3077 | 29.8115 | 16.1667 | 1224 |
| Site 26 | 121.3123 | 29.8091 | 16.1750 | 1221 |
| Site 27 | 121.3120 | 29.8032 | 16.2333 | 1225 |
| Site 28 | 121.3098 | 29.8040 | 16.2333 | 1225 |
| Site 29 | 121.3075 | 29.8034 | 15.9833 | 1246 |
| Site 30 | 121.3044 | 29.8035 | 15.9833 | 1246 |
| Site 31 | 121.3024 | 29.8034 | 15.9833 | 1246 |
| Site 32 | 121.3213 | 29.8073 | 16.2792 | 1220 |
| Site 33 | 121.3181 | 29.8101 | 15.9667 | 1239 |
| Site 34 | 121.3188 | 29.8095 | 15.9667 | 1239 |
| Site 35 | 121.3203 | 29.8104 | 15.9667 | 1239 |
| Site 36 | 121.3217 | 29.8120 | 15.9667 | 1239 |
| Site 37 | 121.3205 | 29.8078 | 16.2792 | 1220 |
| Site 38 | 121.3185 | 29.8025 | 16.2792 | 1220 |
| Site 39 | 121.3165 | 29.7993 | 16.2750 | 1221 |
| Site 40 | 121.3161 | 29.7973 | 16.2750 | 1221 |
| Site 41 | 121.3242 | 29.8039 | 16.2792 | 1220 |
| Site 42 | 121.3253 | 29.8055 | 15.9792 | 1248 |
| Site 43 | 121.3250 | 29.8066 | 15.9792 | 1248 |
| Site 44 | 121.3262 | 29.8074 | 15.9792 | 1248 |
| Site 45 | 121.3265 | 29.8092 | 15.7500 | 1260 |
| Site 46 | 121.2734 | 29.8298 | 15.8167 | 1231 |
| Site 47 | 121.2813 | 29.8288 | 15.9208 | 1228 |
| Site 48 | 121.2871 | 29.8239 | 15.9750 | 1231 |
| Site 49 | 121.2828 | 29.8264 | 15.9208 | 1228 |
| Site 50 | 121.2858 | 29.8204 | 15.9750 | 1231 |
| Site 51 | 121.2687 | 29.7985 | 15.8083 | 1247 |
| Site 52 | 121.2666 | 29.8015 | 15.3500 | 1283 |
| Site 53 | 121.2656 | 29.8049 | 15.3500 | 1283 |
| Site 54 | 121.2664 | 29.8069 | 15.3500 | 1283 |
| Site 55 | 121.2707 | 29.8009 | 15.5792 | 1266 |
| Site 56 | 121.2709 | 29.8008 | 15.5792 | 1266 |
| Site 57 | 121.2797 | 29.7927 | 15.8792 | 1242 |
| Site 58 | 121.2940 | 29.7861 | 16.1625 | 1233 |
| Site 59 | 121.2704 | 29.7962 | 15.8083 | 1247 |
| Site 60 | 121.2875 | 29.7897 | 16.0708 | 1236 |
| Site 61 | 121.2942 | 29.7865 | 16.1625 | 1233 |
| Site 62 | 121.2769 | 29.7691 | 15.6083 | 1273 |
| Site 63 | 121.2733 | 29.7728 | 15.2250 | 1306 |
| Site 64 | 121.2719 | 29.7765 | 14.7792 | 1348 |
| Site 65 | 121.2774 | 29.7630 | 15.6500 | 1274 |
| Site 66 | 121.2740 | 29.7595 | 15.6583 | 1264 |
| Site 67 | 121.2730 | 29.7552 | 15.3083 | 1303 |
| Site 68 | 121.2958 | 29.7486 | 15.9542 | 1258 |
| Site 69 | 121.2960 | 29.7490 | 15.9542 | 1258 |
| Site 70 | 121.2961 | 29.7531 | 16.1292 | 1246 |
| Site 71 | 121.2972 | 29.7538 | 16.1292 | 1246 |
| Site 72 | 121.3052 | 29.7589 | 16.3417 | 1230 |
| Site 73 | 121.3144 | 29.7654 | 16.3833 | 1228 |
| Site 74 | 121.2998 | 29.7672 | 16.2250 | 1235 |
| Site 75 | 121.3072 | 29.7712 | 16.3208 | 1231 |
| Site 76 | 121.3017 | 29.7826 | 16.2542 | 1230 |
| Site 77 | 121.3119 | 29.7799 | 16.3208 | 1231 |
| Site 78 | 121.3093 | 29.7866 | 16.3375 | 1221 |
| Site 79 | 121.3136 | 29.7927 | 16.2750 | 1221 |
| Site 80 | 121.3193 | 29.7827 | 16.3625 | 1231 |
| Site 81 | 121.3349 | 29.7753 | 16.3458 | 1234 |
| Site 82 | 121.3372 | 29.7761 | 16.3458 | 1234 |
| Site 83 | 121.3422 | 29.7733 | 16.4750 | 1240 |
